# A new human autologous hepatocyte/macrophage co-culture system that mimics drug-induced liver injury–like inflammation

**DOI:** 10.1007/s00204-024-03943-8

**Published:** 2024-12-22

**Authors:** Andrea Zimmermann, Andrea Scheffschick, René Hänsel, Hannes Borchardt, Jia Li Liu, Sabrina Ehnert, Gerda Schicht, Lena Seidemann, Achim Aigner, Susanne Schiffmann, Andreas Nüssler, Daniel Seehofer, Georg Damm

**Affiliations:** 1 Department of Hepatobiliary Surgery and Visceral Transplantation, Clinic and Polyclinic for Visceral, Transplant, Thoracic and Vascular Surgery, Leipzig University Medical Center, Leipzig, Germany; 2 Saxonian Incubator for Clinical Translation (SIKT), University of Leipzig, Leipzig, Germany; 3 Institute for Medical Informatics, Statistics and Epidemiology (IMISE), Leipzig University, Leipzig, Germany; 4 Rudolf-Boehm-Institute for Pharmacology and Toxicology, Clinical Pharmacology, Faculty of Medicine, University of Leipzig, Leipzig, Germany; 5 Department of General, Visceral- and Transplantation Surgery, Charité - University Medicine Berlin, Berlin, Germany; 6 Department of Traumatology, BG Trauma Center, University of Tübingen, Tübingen, Germany; 7 Fraunhofer Institute for Translational Medicine and Pharmacology ITMP, Frankfurt Am Main, Germany

**Keywords:** Drug-induced liver injury, Primary human macrophages, Primary human hepatocytes, Co-culture models

## Abstract

The development of in vitro hepatocyte cell culture systems is crucial for investigating drug-induced liver injury (DILI). One prerequisite for monitoring DILI related immunologic reactions is the extension of primary human hepatocyte (PHH) cultures towards the inclusion of macrophages. Therefore, we developed and characterized an autologous co-culture system of PHH and primary human hepatic macrophages (hepM) (CoC1). We compared CoC1 with a co-culture of the same PHH batch + M0 macrophages derived from THP1 cells (CoC2) in order to represent a donor independent macrophage reaction. Then, we treated the mono- and co-cultures with drugs that cause DILI—menadione (MEN, 1 or 10 µM, 3 h), diclofenac (DIC, 0.5 or 5 mM, 6 h), or acetaminophen (APAP, 0.5 or 5 mM, 6 h)—and assessed culture stability, cell activity, macrophage differentiation, cytokine production and cell viability. Without drug treatment, CoC1 was the most stable over a culture time of up to 60 h. Cytokine array analysis revealed a proinflammatory profile of PHH mono-cultures due to isolation stress but showed different influences of hepM and M0 on the cytokine profile in the co-cultures. MEN, DIC and APAP treatment led to donor-dependent signs of cell stress and toxicity. HepM can either promote or reduce the DILI effects donor dependently in CoC1. CoC2 are slightly less sensitive than CoC1 in representing DILI. In summary, we present a new autologous co-culture system that can mimic DILI in a donor-dependent manner. This cellular system could be useful for new drug testing strategies and reducing animal testing.

**Supplementary Information:**

The online version contains supplementary material available at 10.1007/s00204-024-03943-8.

## Introduction

The liver is the largest solid organ in the body and plays a central role in xenobiotic metabolism, including the biotransformation of drugs (Almazroo et al. 2017; Trefts et al. 2017). Xenobiotics or other factors like viruses or alcohol may induce liver injury. In so-called drug-induced liver injury (DILI), drug side effects lead to liver damage that may result in hospitalization or even death (Andrade et al. 2019; Abid et al. 2020). The main event in DILI is the death of hepatocytes, the parenchymal cells of the liver that harbor drug-metabolizing enzymes and are highly metabolically active. Metabolism of drugs can lead to the formation of reactive metabolites, which make hepatocytes prone to DILI. In this context, menadione (MEN), acetaminophen (APAP), and diclofenac (DIC) represent well-studied drugs that can cause DILI. This phenomenon can be differentiated into a dose-dependent form called intrinsic DILI and an unpredictable form called idiosyncratic DILI. Compounds that cause intrinsic DILI, such as MEN and APAP, show direct hepatotoxic effects by inducing reactive oxygen intermediates (ROI) as a result of redox cycling of reactive metabolites (Badr et al. 1987). In the case of DIC, reactive metabolites can bind to proteins, leading to the formation of neo-haptens, which are targets for immune cells, and resulting in sensitizing reactions (Badr et al. 1987; Aithal 2004; Yoon et al. 2016).

The liver harbors a multitude of immune cells, which are part of the non-parenchymal liver cell (NPC) compartment. In general, NPC consisting of cholangiocytes, endothelial cells, stellate cells, macrophages, and various lymphocyte populations, support the functions of hepatocytes. Hepatic macrophages (hepM) are the most abundant immune cells in the liver and comprise Kupffer cells, the resident liver macrophages and the predominant macrophage population in this organ, and monocyte-derived macrophages (Wen et al. 2021). Via the portal vein, hepM are constantly in contact with substances from the intestine, such as food components, bacterial products, or dead and dying cells, and can detect and eliminate them by phagocytosis (Dixon et al. 2013). Activation of hepM resulting in the secretion of cytokines is essential for the liver’s response to infection, inflammation, and injury. Different pathological conditions can favor different developmental stages of macrophages. RNA sequencing (RNAseq) analysis revealed that regulatory or inflammatory macrophages exist in the liver (MacParland et al. 2018). HepM exert a high tolerance to endogenous tissue in the healthy liver to avoid autoimmune responses. Loss of this tolerance can lead to liver injury.

Hepatocyte monoculture is the gold standard for studying the in vitro hepatotoxicity of drugs (Tasnim et al. 2021). However, after initial minor damage to hepatocytes by drugs, a secondary reaction may occur involving inflammatory processes based on NPC (Granitzny et al. 2017; Ganey et al. 2004). HepM are known to be involved in DILI, and their activation may result in the secretion of pro- or anti-inflammatory cytokines (Ito et al. 2003; Dugan et al. 2011). Additionally, many toxicities observed in vivo cannot be replicated in vitro, or only to a limited extent (Schyschka et al. 2013). Immune cells that cause or contribute to DILI may play a central role in this regard. Rats and mice provide animal models that are essential in toxicology to study DILI mechanisms involving the immune system in vivo. However, not every drug that induces DILI in humans also induces DILI in animals and vice versa, suggesting that different mechanisms may induce DILI in different species. In mouse studies, hepM have been shown to have both protective and deleterious functions in APAP-induced DILI (Ito et al. 2003; Ju et al. 2002). The partially contradictory results for the role of hepM in DILI and the question of transferability of the results to humans highlights the need to use human cellular systems to study DILI mechanisms.

Many human cell studies have used DILI-associated cytokines, the conditioned medium of immune cells, human cell lines, or co-culture systems of primary cells to investigate the immune-mediated effects of DILI on hepatocytes (Tasnim et al. 2021). However, there are no co-culture systems consisting of autologous primary human hepatocytes (PHH) and primary human macrophages to analyze the immune-mediated effect of drugs, an approach that would represent the in vivo situation of DILI in humans. Kegel et al. 2015 showed that autologous hepM treated with the conditioned medium of hepatocytes incubated with hepatotoxic drugs can induce hepatocyte stress or damage. Here, we sought to follow up on this approach by performing co-cultures of autologous PHH and hepM to study DILI mechanisms. One challenge when working with primary cell cultures is the standardization of the experiments due to strong (genetic) variability between donors. Using macrophages derived from a monocyte cell line such as THP1 could prevent these variations. Therefore, we aimed to compare PHH and hepM co-cultures with PHH and differentiated THP1 cell co-cultures in terms of their ability to represent DILI effects. We treated these co-cultures with hepatotoxic drugs exemplified by MEN, APAP, or DIC, and analyzed hepatotoxicity based on cell activity, ROI formation, cell death, and the inflammatory reaction. We found that PHH and macrophage co-cultures could be used to analyze drug-induced immune cell–mediated hepatotoxicity in the early phase of drug development**.**

## Results

### Description of the study design

**Fig. 1:**
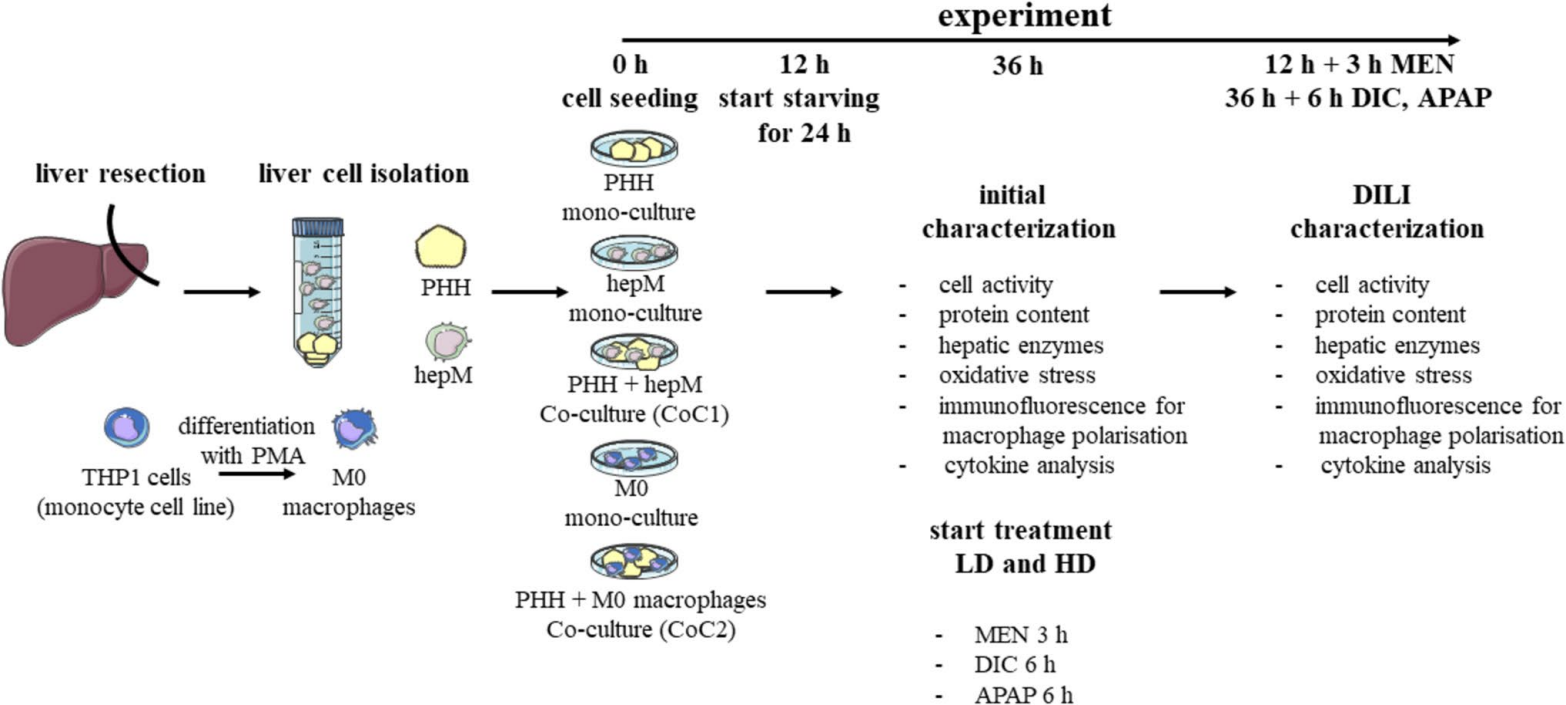
Experimental setup for the drug-induced liver injury (DILI) experiments. THP1 cells were stimulated with 10 ng/mL phorbol-12-myristate-13-acetate (PMA) for differentiation into M0 macrophages for 24 h, followed by culture in medium for another 24 h prior to the co-culture experiments. Primary human hepatocytes (PHH) and hepatic macrophages (hepM) were isolated from the same liver tissue samples. All cells were seeded into mono- and co-cultures and grown overnight (12–16 h). Then, the cells were starved for 24 h. After a total time of 36 h, the cultures were characterized initially. Thereafter, the cultures were treated with a low or high dose (LD or HD, respectively) of menadione (MEN, 1 or 10 µM, 3 h), diclofenac (DIC, 0.5 or 5 mM, 6 h), or acetaminophen (APAP, 0.5 or 5 mM, 6 h). Then, the cultures were characterized to assess DILI. CoC1 = co-culture of PHH + hepM; CoC2 = co-culture of PHH + M0 macrophages differentiated from THP1 cells. The illustration has been generated using Servier Medical Art

We investigated the role of human macrophages in DILI by co-culturing PHH with either hepM isolated from the same liver tissue sample or with THP1 cells differentiated to M0 macrophages. This approach yielded standardized results and thus allowed us to compare the different co-culture systems. We co-cultured PHH and macrophages at a ratio of 4:1, which corresponds to the ratio of the cells in the healthy liver (Lopez et al. 2011). To induce DILI, we treated the co-cultures with MEN, APAP, or DIC. From our experience hepatotoxic effects of a specific compound show a wide variability in primary human cells. Therefore, using a classic EC50 approach often fails and we decided to use subtoxic concentrations of our test compounds. We evaluated concentration- and time-dependent toxic effects (Fig. S1A–C) to determine a high dose that produced subtoxic effects, a low dose an order of magnitude lower, and the appropriate incubation times. Specifically, we used the following treatments: 1 or 10 µM MEN for 3 h, 0.5 or 5 mM DIC for 6 h, and 0.5 or 5 mM APAP for 6 h. Furthermore, we examined the time-dependent toxic effects of phorbol-12-myristate-13-acetate (PMA), which we used to differentiate THP1 cells to M0 macrophages (Fig. S2A–F). Because incubation with PMA for ≥ 48 h was toxic to THP1 cells, we used a PMA incubation time of 24 h followed by a regeneration phase of 24 h. To avoid the influence of growth factors, hormones, or other compounds that could interfere with DILI detection, we performed the experiments in starvation medium devoid of fetal calf serum (FCS), insulin, and dexamethasone. The resulting study design is shown in Fig. 1. We used cells from various donors to establish and characterize the experimental setup as well as the final application. The donor characteristics are shown in Table 1.

**Table 1:** Allocation of donors to the experiments performed. The table show clinical data of the 14 patients from which liver tissue samples were obtained

Experiment	Donor ID	Sex	Age	Diagnosis (final)	BMI	chemo-therapy
characterization CoC1 (with hepM)	D01*	m	71	Intrahep. Cholangiocarcinoma	24	No
characterrization CoC1 (with hepM)	D02	f	58	Colorectal liver metastasis	26	No
characterization CoC1 (with hepM)	D03	m	47	Colorectal liver metastasis	24	No
characterization CoC2 (with M0)	D04**	f	39	Focal nodular hyperplasia	20	No
characterization CoC2 (with M0)	D05	m	68	Adenocarcinoma common hepatic duct	30	No
characterization CoC2 (with M0)	D06	f	71	Moderately differentiated intrahepatic adenocarcinoma	18	No
Concentration finding (Tox)	D07	f	71	Intrahep. Cholangiocarcinoma	26.2	Unknown
Concentration finding (Tox)	D08	m	56	Neuroendocrine tumor	24.2	Unknown
Culture Morphology (CoC1)	D09	f	45	Polycystic liver disease	30	No
Tox (CoC1 + CoC2)	D10	m	29	Colorectal liver metastasis, adenocarcinoma	22	Yes
Tox (CoC1 + CoC2)	D11	m	31	Colorectal liver metastasis, adenocarcinoma	28	Yes
Tox (CoC1 + CoC2)	D12*	m	71	Intrahepatic cholangiocarcinoma	24	No
Culture Morphology (CoC1)	D13	f	54	Hemangioma	26.6	No
Culture Morphology (CoC2)	D14**	f	39	Focal nodular hyperplasia	20	No
Culture Morphology (CoC1 + CoC2)	D15	f	44	Adenoma	24.5	No
Culture Morphology (CoC2)	D16	f	51	Colorectal liver metastasis	23.5	Yes

* and ** mark identical donors used in different experiments

### Characterization of PHH and macrophage mono- and co-cultures confirms their stability over time, which makes them suitable for DILI experiments

**Fig. 2:**
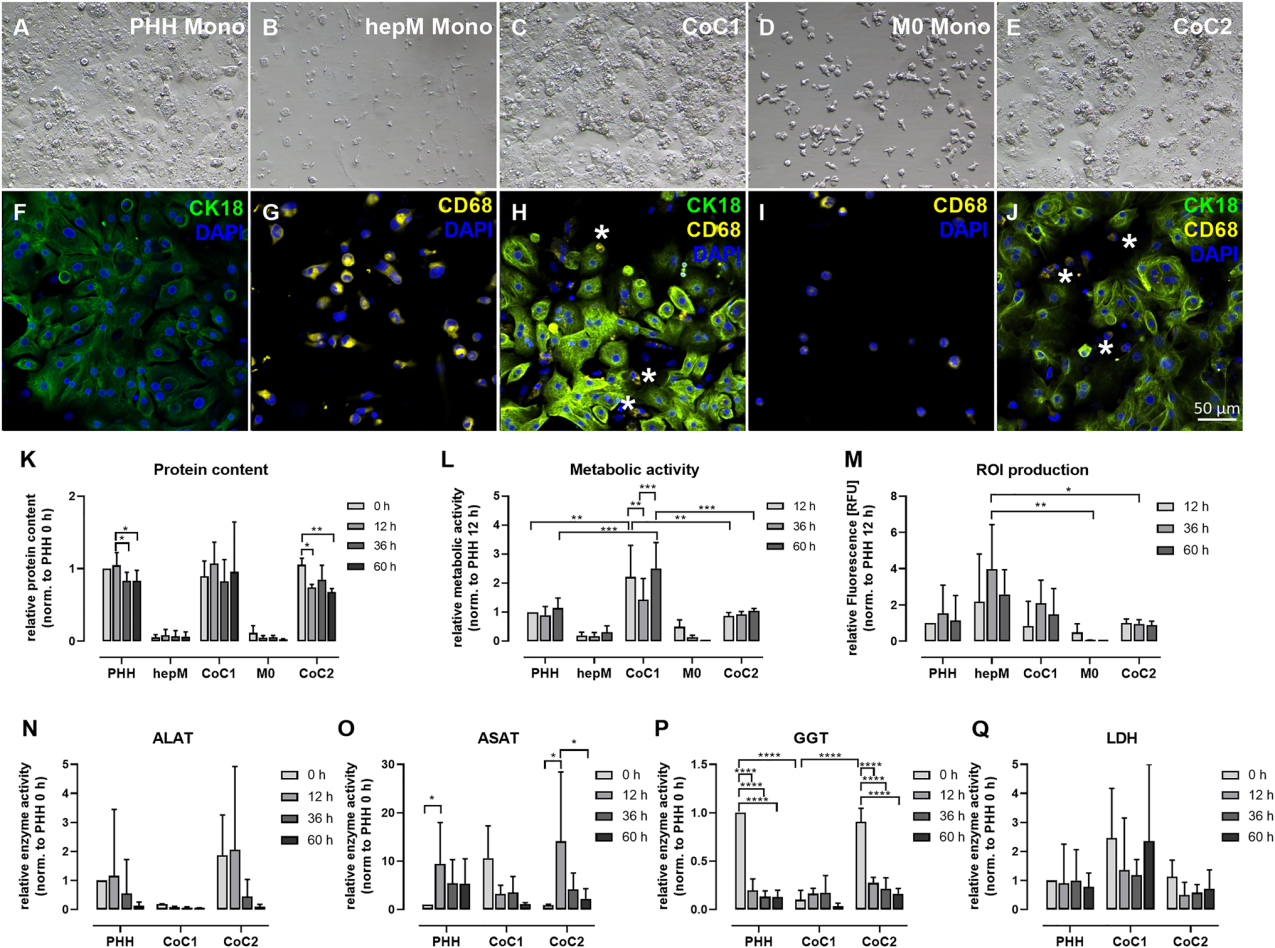
Light microscopy and immunofluorescence images of mono– and co-cultures and characterization of the cultures. Light microscopy and immunofluorescence images of (**A**, **F**) primary human hepatocyte (PHH) monocultures, (**B**, **G**) hepatic macrophage (hepM) monoculture, (**C**, **H**) PHH + hepM co-culture (CoC1), (**D**, **I**) M0 macrophage monocultures, and (**E**, **J**) PHH + M0 macrophage co-culture (CoC2) after culturing for 36 h. In the immunofluorescence images, CD68 (yellow) represents macrophages, CK18 (green) represents cytokeratin 18 in PHH, and DAPI (blue) shows nuclei. The cultures were characterized with regard to (**K**) the protein content using the bicinchoninic acid (BCA) protein assay; (**L**) metabolic activity with the XTT assay; (**M**) reactive oxygen intermediate (ROI) production with the DCF assay; and (**N**) alanine aminotransferase (ALAT), (**O**) aspartate aminotransferase (ASAT), (**P**) gamma-glutamyl transferase (GGT), and (**Q**) lactate dehydrogenase (LDH) activity. The asterisks indicate CD68-positive macrophages. The data represent at least three independent biological triplicates. For characterization of CoC1, cells derived from donors 1–3 were used. For characterization of CoC2, cells derived from donors 4–6 were used. For characterization of PHH monocultures, cells derived from donors 1–6 were used. * p < 0.05, ** p < 0.01, *** p < 0.001, **** p < 0.0001

Light and fluorescence microscopy of the mono- and co-cultures at 36 h (the time point of initial characterization) revealed the arrangement of PHH and immune cells in the co-cultures (Fig. 2A–J**)**. The PHH monocultures showed high confluency and the typical morphonology of stretched hepatocytes with prominent nuclei (Fig. 2A and F). The hepM monocultures presented a heterogeneous cell population varying widely in cell size and shape (Fig. 2B and G). In contrast, the THP1-derived M0 macrophage monocultures showed a more homogeneous culture of macrophages with a spherical shape and prominent filopodia. Compared to the hepM culture, the macrophages were at the upper end of the size distribution (Fig. 2D and I). In the PHH + hepM (CoC1) and PHH + M0 macrophage (CoC2) co-cultures, hepM and M0 were located adjacent to PHH or attached to PHH (Fig. 2C, H, E, and J). Macrophages located adjacent to PHH were mostly spherical in shape and had a round nucleus. Macrophages located between two PHH had an elongated morphology with a stretched nucleus. Timelapse videos of both co-cultures showed static PHH and dynamic macrophages. However, hepM in CoC1 showed a higher level of mobility compared with the M0 macrophages in CoC2 (Fig. S3 and Supplementary Files 2, 3, 4, 5). Furthermore, timelapse videos showed two different types of macrophages in terms of shape and mobility. Spherical macrophages stayed stationary and adjacent to PHH, but they showed highly active filopodia. Stretched macrophages showed migratory behavior and some traversed long distances. There were a particularly high number of migratory macrophages from cells isolated from liver tissue samples derived from patients after portal vein embolization (CoC1 of D09, Supplementary File 8).

We also characterized the untreated co-cultures with regard to cell activity, the protein level, ROI production, and cell death. We used an incubation time of up to 60 h, covering the 36 h initial regeneration phase and a 24 h potential application time. Over this time, the protein content of CoC1 was more stable but showed large variations, indicating a donor-specific influence of hepM. In contrast, the PHH monocultures and CoC2 showed a slightly reduced protein content over time (Fig. 2K). CoC1 showed higher cell activity compared with the PHH monocultures and CoC2 (Fig. 2L). There was a decrease after 36 h, followed by an increase after 60 h. We observed a similar trend in the protein content, suggesting an influence of hepM on hepatocyte protein and metabolic activity. ROI production was highest in the hepM monocultures, with no difference in the ROI level over the cultivation period (Fig. 2M). To assess cell death, we measured the activity of alanine aminotransferase (ALAT), aspartate aminotransferase (ASAT), gamma-glutamyl transferase (GGT), and lactate dehydrogenase (LDH) in the cell culture supernatant over time (Fig. 2N–Q). ALAT and GGT activities were high at the beginning (0 h). While GGT activities showed a sharp decrease after the adherence phase (0 h), ALAT activities decrease over time, suggesting that the cells were stressed from the isolation procedure and/or the initial cultivation (Fig. 2N and P). ASAT activity was high at 12 h but decreased thereafter, and LDH activity was relatively stable over the entire culture period (Fig. 2O and Q). Again, CoC1 showed the highest LDH activity and high variability. Taken together, the cultures were stable over a period of 60 h and based on cell recovery after cell isolation, we considered 36 h after isolation to be a good starting point for treatment. In general, the cultures are suitable to characterize DILI.

### PHH and macrophage co-cultures display a distinct inflammatory status compared with the monocultures

**Fig. 3:**
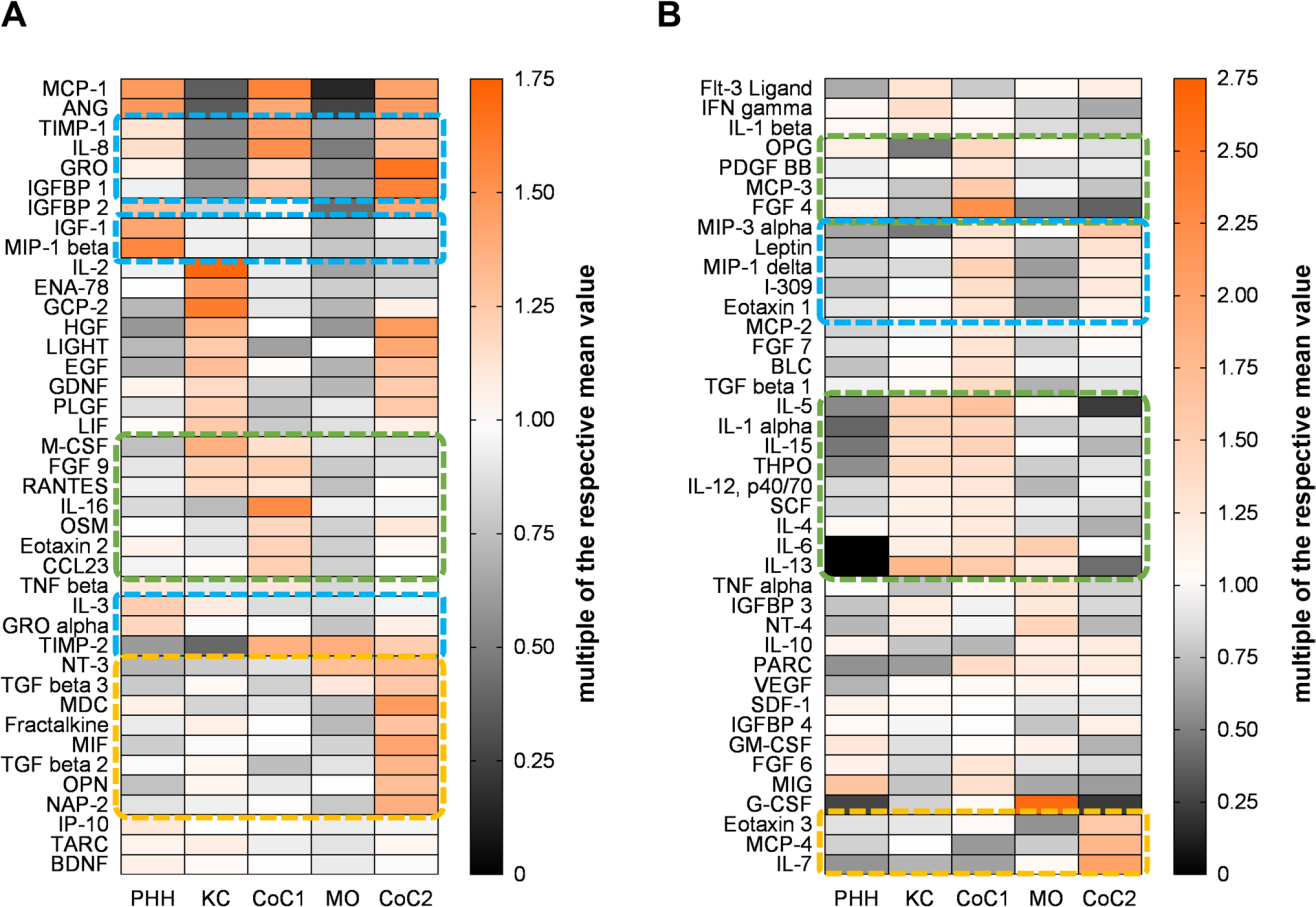
Analysis of cytokines, chemokines and growth factors in steady-state cultures. The levels of cytokines, chemokines and growth factors in the steady-state cultures without drug treatment were analyzed with a protein array after co-culture for 36 h. (**A**) Protein array with high expression profiles and (**B**) with low expression profiles. The blue boxes indicate targets with expression changes in both co-cultures compared with the PHH monocultures. The green and yellow boxes indicate targets with expression changes for either CoC1 (green) or CoC2 (yellow) compared with the PHH monocultures. The data represent three pooled biological triplicates of donors 10, 11, and 12. PHH = primary human hepatocytes; hepM = hepatic macrophages; CoC1 = co-culture of PHH + hepM; CoC2 = co-culture of PHH + M0 macrophages

Next, we investigated the basal inflammatory status of the cultures by using a cytokine protein array (Fig. 3). Some classical pro- and anti-inflammatory cytokines, including tumor necrosis factor alpha (TNF-α), interleukin 10 (IL-10), and transforming growth factor beta 1 (TGF-β1), showed low levels; hence, they should be interpreted with caution (Fig. 3B). We identified distinct sets of cytokines or chemokines that were elevated or reduced in the mono- and co-cultures (Fig. 3A). Some cytokines, such as IL-8, were elevated in both CoC1 and CoC2 compared with the PHH monocultures. Some cytokines, such as macrophage inflammatory protein 1beta (MIP-1β) and chemokine (C-X-C motif) ligand 1 (CXCL1, also known as GROα), were reduced in CoC1 and CoC2 compared with the PHH monocultures. IL-16, chemokine (C–C motif) ligand 23 (CCL23), and CCL24 (also known as eotaxin-2) were elevated in CoC1 compared with the PHH monocultures and CoC2, while the cytokine levels were low in the hepM monocultures. Thus, IL-16, CCL23, and CCL24 were elevated in the co-culture but not in hepM and PHH separately. Macrophage-derived chemokine (MDC, also known as CCL22), TGF-β2, and TGF-β3 were upregulated in CoC2 compared with the PHH monocultures and CoC1, but again TGF-β2 and TGF-β3 were low in the M0 monocultures. Furthermore, hepatocyte growth factor (HGF) and epidermal growth factor (EGF) were higher in the hepM monocultures than in CoC1. Hence, the pro-regenerative state of hepM is not transferred to the autologous co-cultures. In contrast, a pro-regenerative state was induced in CoC2 even though these growth factors were absent in their corresponding M0 monocultures. In general, both CoC1 and CoC2 showed higher or different levels of both anti- and pro-inflammatory cytokines compared with the PHH monocultures, suggesting that the interplay of hepM or M0 macrophages with PHH in the co-culture can influence the cytokine profile. The inflammatory status of CoC1 and CoC2 cannot be clearly determined because both pro- and anti-inflammatory cytokines are upregulated.

### Analysis of metabolic activity in treated and untreated cultures reveals that DILI effects are donor dependent

We assessed hepatoxicity in the PHH monocultures, CoC1, and CoC2 with and without drug treatment by examining mitochondrial activity with the XTT assay; this approach provides an indication of cell viability. A decrease in XTT reduction following treatment indicates a potential hepatotoxic effect. We used MEN, APAP, and DIC as model hepatotoxic drugs at low and high doses, both in the subtoxic range. We investigated the metabolic activity in cells derived from three different donors (donors 10–12, Table 1). Donors 10 and 11 were patients with colorectal liver metastasis who had received chemotherapy. Although they did not have primary liver disease, their liver tissue cannot be regarded as completely healthy. Donor 12 had intrahepatic cholangiocarcinoma, which usually develops on the background of chronic liver disease.

**Fig. 4:**
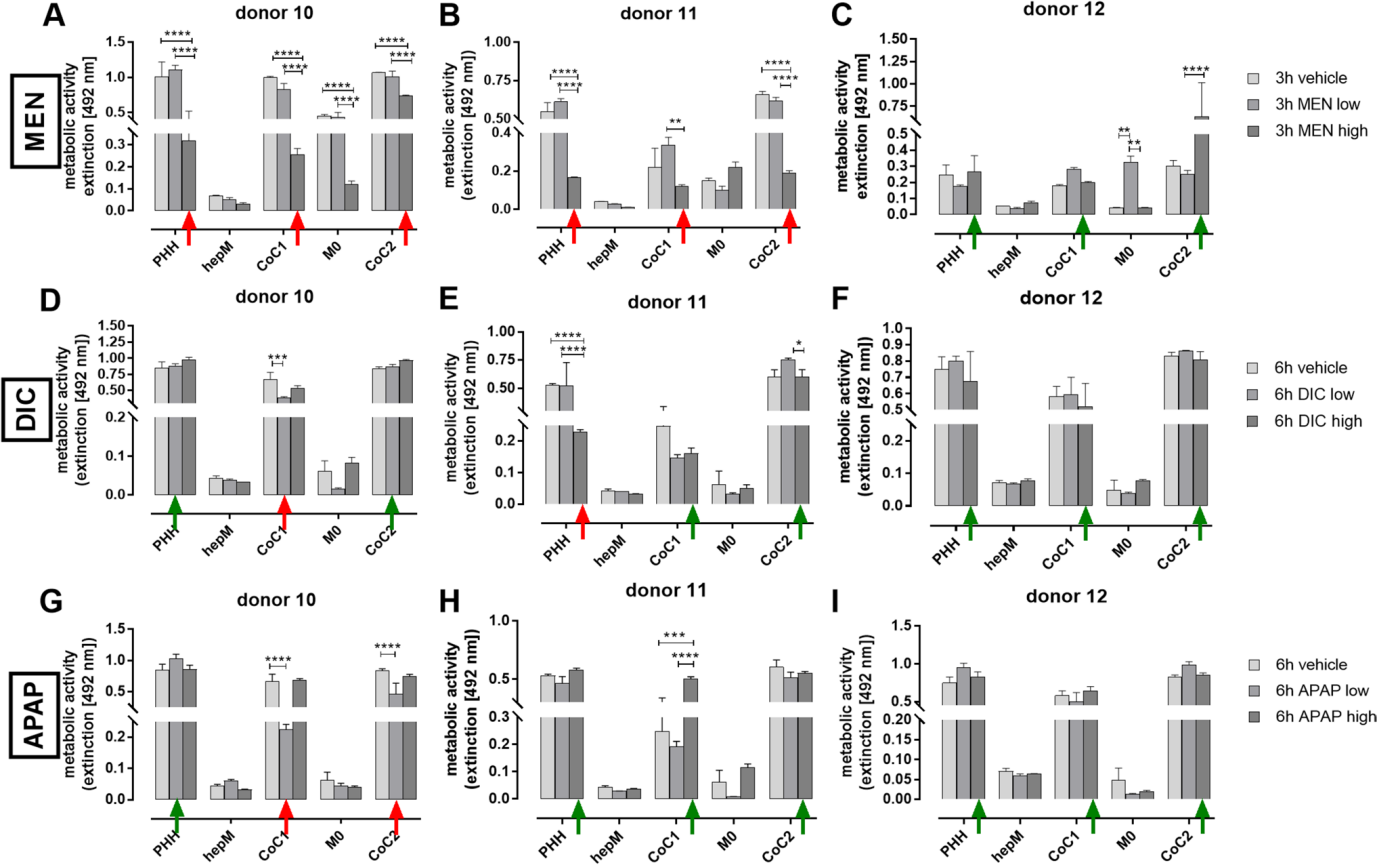
Treatment of mono– and co-cultures with MEN, DIC and APAP and analysis of metabolic activity. After cultivation for 36 h, the cultures were treated with low- or high-dose (**A–C**) menadione (MEN) for 3 h, (**D–F**) diclofenac (DIC) for 6 h, or (**G–I**) acetaminophen (APAP) for 6 h. Metabolic activity was analyzed using the XTT assay to assess hepatotoxicity. The red arrows indicate a reduction in metabolic activity and hence hepatotoxic effects. The green arrows indicate no reduction in metabolic activity and hence no hepatotoxic effect. The data represent three technical triplicates of three donors. Liver cells from donors 10, 11, and 12 were used for the experiments. PHH = primary human hepatocytes; hepM = hepatic macrophages; CoC1 = co-culture of PHH + hepM; CoC2 = co-culture of PHH + M0 macrophages. * p < 0.05, ** p < 0.01, *** p < 0.001, **** p < 0.0001

CoC1 with cells derived from donors 10 and 11 showed toxic effects when treated with MEN compared with the vehicle control (Fig. 4A and B). In contrast, cultures with cells derived from donor 12 reacted differently to those from donors 10 and 11 (Fig. 4C, F, and I). There were no signs of reduced activity in the PHH mono- or co-cultures compared with the vehicle control, suggesting higher tolerance. Regarding DIC and APAP treatment, CoC1 with cells derived from donor 10 showed decreased metabolic activity following low-dose DIC or APAP treatment compared with the vehicle control; there was no effect in the PHH monoculture from donor 10 (Fig. 4D and G). In addition, CoC2 with cells derived from donor 10 showed reduced metabolic activity compared with the PHH monoculture after low-dose APAP treatment (Fig. 4D and G). For donor 11, we noted reduced metabolic activity in the PHH monoculture after high-dose DIC treatment. Although DIC treatment reduced metabolic activity in CoC1 with cells derived from donor 11, the difference was statistically not significant. Moreover, APAP treatment did not affect CoC1, and DIC or APAP treatment did not affect CoC2 (Fig. 4E and H). Notably, compared with low-dose APAP, high-dose APAP treatment increased metabolic activity in CoC1 with cells from donor 10 or 11. Presumably, the higher stress level leads to mutual activation of hepM and PHH. Our results indicate that metabolic activity can show a decrease classically interpreted as hepatotoxicity but also an increase probably as an indicator of activation. Therefore, metabolic activity is an unreliable marker for investigating viability in PHH and macrophage co-cultures.

### Cell death is not significantly induced in the cultures following MEN, DIC, and APAP treatment

**Fig. 5:**
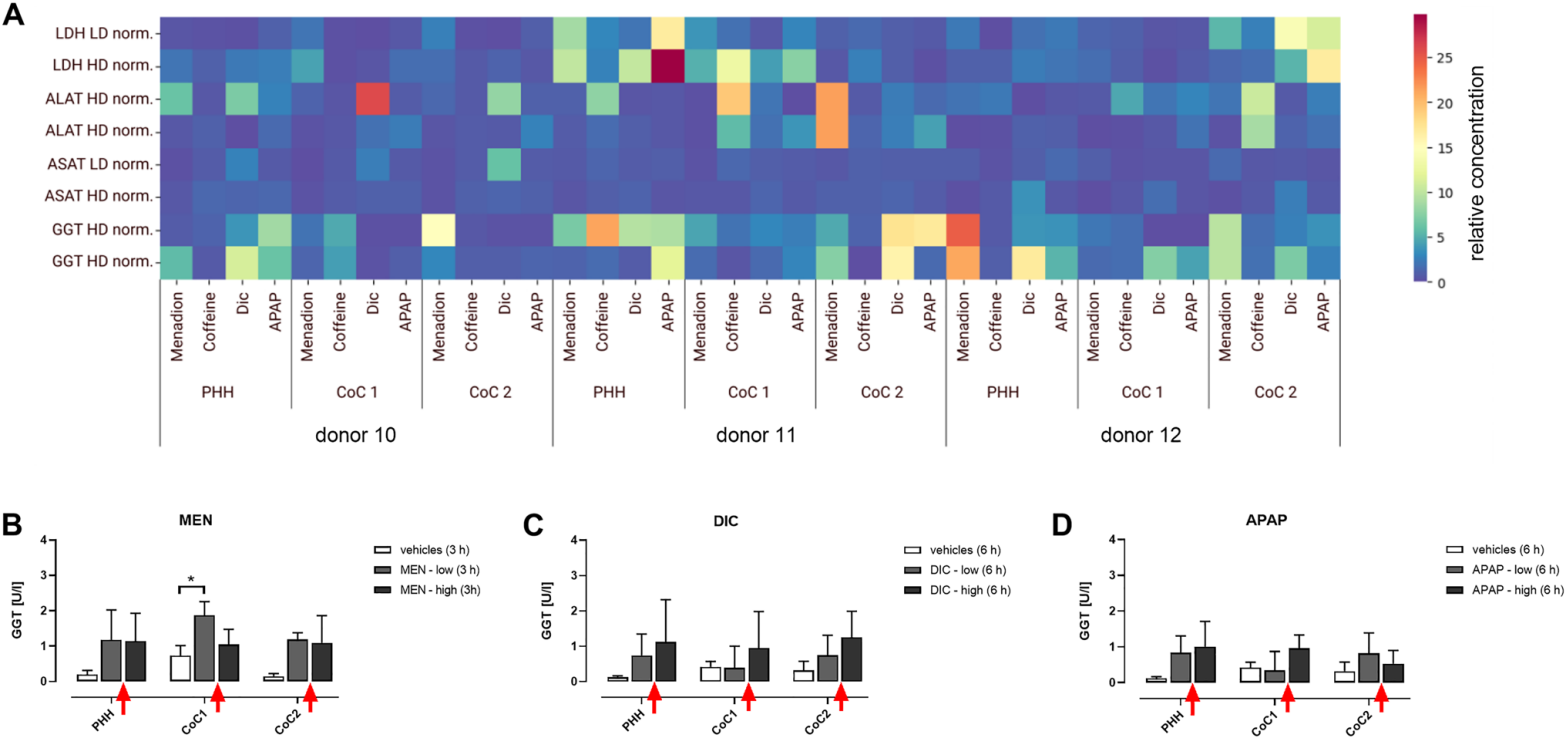
Analysis of LDH, ALAT, ASAT, and GGT following MEN, DIC and APAP treatment to determine necrosis in the cultures. **(A**) The heat map shows the influence of MEN, DIC, and APAP in a donor-dependent manner (10, 11, and 12) on the enzymes lactate dehydrogenase (LDH), alanine aminotransferase (ALAT), aspartate aminotransferase (ASAT), and Gamma-glutamyl transferase (GGT). Blue indicates low expression of the enzymes. Red indicates high expression of the enzymes. GGT was measured in the cell culture supernatant to assess necrosis following (**B**) menadione (MEN), (**C**) diclofenac (DIC), or (**D**) acetaminophen (APAP) treatment of the cultures derived from donors 10, 11, and 12 combined. The red arrows indicate an increase in GGT activity compared with the vehicle control. The data represent three independent biological triplicates. * p < 0.05

Our data showed an effect of hepM and M0 macrophages on metabolic activity in drug-treated co-cultures, raising the question of whether there was reduced viability. To characterize potential cell death, we investigated markers for necrosis, apoptosis, and cell stress in cultures with and without drug treatment. To assess cell death by necrosis, we analyzed membrane integrity based on the release of LDH, ALAT, ASAT, and GGT into the culture supernatants (Figs. 5 and S4). Due to their high expression in hepatocytes, ALAT, ASAT, and GGT are liver specific. ALAT and ASAT are found inside hepatocytes and are therefore only measured in elevated concentrations in the event of severe hepatocyte damage. GGT, however, is bound to the membrane of liver cells and can increase even in the case of mild hepatocyte damage. During the culture, hepatic transferases were not released from hepM and M0 monocultures, proving that these enzymes are hepatocyte-specific markers (Fig. S4). In contrast, LDH was released from the PHH, hepM, and M0 monocultures, indicating that this enzyme can be released from hepatocytes and macrophages. In general, the enzyme activities were increased moderately up to 36 h of culture, probably due to initial isolation stress. Afterwards, the PHH monocultures were stable while the M0 monocultures showed somewhat high LDH release. Overall, the co-cultures were more stable than the monocultures during the entire cultivation.

We considered LDH and GGT to be the most sensitive markers for the cell death analysis based on their release from the drug-treated PHH monocultures (Fig. 5A). The fact that ALAT and ASAT activities were rarely increased after DIC or APAP treatment of the PHH monocultures confirmed that we used subtoxic concentrations of these drugs. Treatment with MEN increased GGT activity compared with the vehicle control in the PHH monocultures, CoC1, and CoC2 from all three donors combined (Fig. 5B). DIC or APAP treatment induced dose-dependent GGT release in PHH monocultures, CoC1, and CoC2 (Fig. 5C and D). There was noteworthy LDH release in the drug-treated PHH monoculture derived from donor 11; it correlated with the GGT release. In addition, drug-treated CoC1 showed increased LDH activity without elevated transferase activities, indicating a certain degree of macrophage stress. Hence, treatment with compounds that can cause DILI resulted in the slight release of either LDH or GGT independent of the co-culture with macrophages, indicating effects between cell stress and mild hepatotoxicity. Consequently, macrophages did not induce cell death by necrosis in the drug-treated co-cultures.

To further characterize the induction of cell stress and cell death by apoptosis, we investigated poly(ADP-ribose)-polymerase 1 (PARP1) expression and cleavage by Western blotting. PARP1 is involved in DNA repair and is upregulated upon cell stress. Upon apoptosis induction, PARP1 is cleaved by caspases. Due to restrictions in the available samples, we could only perform Western blotting successfully for MEN-, DIC-, and APAP-treated cultures derived from donor 11 (Fig. S5A–C). Data analysis revealed neither induction of PARP1 expression nor elevated PARP1 cleavage relative to uncleaved PARP1 (> twofold increase) in the drug-treated PHH monocultures. In contrast, in CoC1 treated with high-dose APAP and CoC2 treated with low-dose DIC a tendency towards elevated PARP1 expression was found. In addition, there were elevated levels of cleaved PARP1 relative to PARP1 in CoC2 treated with high-dose MEN or high-dose DIC (Fig. S5A–C).

Taken together, our data revealed elevated cell stress in drug-treated cultures, denoted by increased GGT release. We confirmed this cell stress in co-cultures derived from donor 11 to be based on the induction of PARP1 expression. Our results did not reveal a clear induction of necrosis or apoptosis by the tested drugs and concentrations. This suggests that the observed reduction in metabolic activity in the MEN-, DIC-, and APAP-treated co-cultures is linked to macrophage-induced cell stress rather than hepatocyte cell death.

### Donor-dependent effects on cytokine regulation could explain DILI effects in the co-cultures

**Fig. 6:**
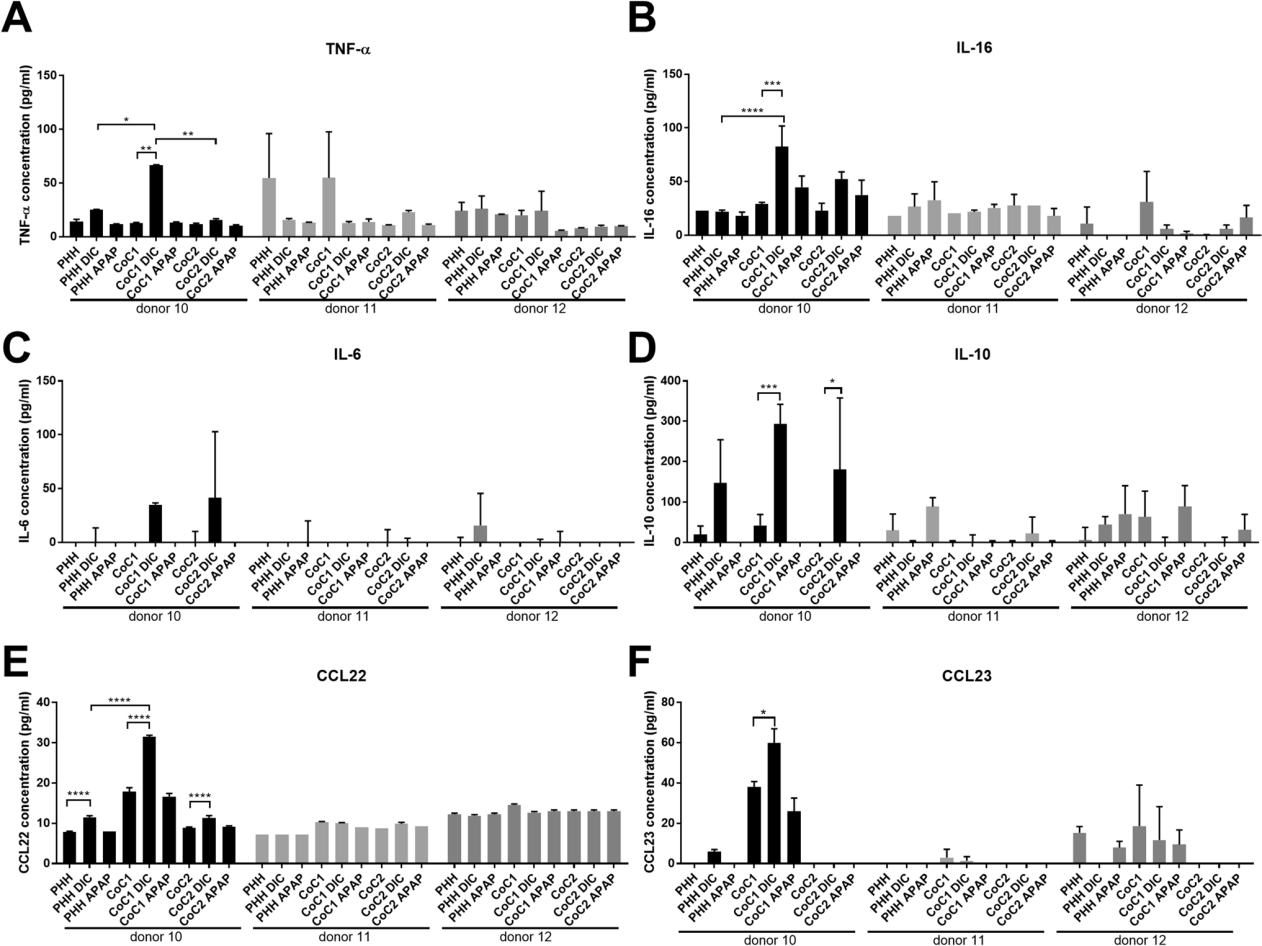
Analysis of cytokines and chemokines in mono- and co-cultures following DIC and APAP treatment. Enzyme-linked immunosorbent assay analysis of (**A**) tumor necrosis factor alpha (TNF-α), (**B**) interleukin 16 (IL-16), (**C**) IL-6, (**D**) IL-10, (**E**) chemokine (C–C motif) ligand 22 (CCL22), and (**F**) chemokine (C–C motif) ligand 23 (CCL23) after low-dose diclofenac (DIC) or acetaminophen (APAP) treatment. The data represent three pooled technical triplicates for each donor (10, 11, and 12) measured in duplicate. PHH = primary human hepatocytes; hepM = hepatic macrophages; CoC1 = co-culture of PHH + hepM; CoC2 = co-culture of PHH + M0 macrophages. Asterisks indicate when DIC or APAP treatment significantly upregulated the cytokine or chemokine: * p < 0.05, ** p < 0.01, *** p < 0.001, **** p < 0.0001

To analyze the influence of drug treatment on DILI-relevant cytokines—and, thereby, to identify potential DILI mechanisms/drivers—we used enzyme-linked immunosorbent assays (ELISAs) to measure the pro- and anti-inflammatory cytokines TNFα, IL-16, IL-6, IL-10, CCL22, and CCL23, which are involved in DILI, liver regeneration, or liver injury in the cultures with and without drug treatment. We analyzed the three donors separately to identify potential donor-dependent effects. The drug-treatment effects on the hepM and M0 monocultures were very limited (Fig. 6). TNF-α was the only cytokine that showed a significant increase in the hepM monoculture from donor 12 after treatment with DIC (Fig. S6A). Surprisingly, DIC or APAP treatment could lead to single cases of cytokine secretion in different batches of THP1-derived M0 macrophages (Fig. S6). The basal levels of DILI-relevant cytokines were very low in the PHH monocultures (Fig. 6A–F). Treatment of the PHH monocultures and the co-cultures from the three donors led to varying effects on the cytokine levels. Cells derived from donor 10 showed significant upregulation of cytokines following DIC treatment, while cells derived from donors 11 and 12 did not. For donor 10, DIC treatment increased all investigated cytokines in CoC1 compared with the vehicle control and the DIC-treated PHH monoculture. This effect is consistent with the reduced metabolic activity (Figs. 4D and 6A, B). We observed a comparable pattern for CoC2 after DIC treatment, but most of the cytokines remained at the same level as observed in the DIC-treated PHH monocultures. For donor 11, TNF-α was the only cytokine elevated in the PHH monoculture and CoC1, suggesting a slightly pro-inflammatory state of these cultures. Drug treatment did not elevate TNF-α further, consistent with the absence of negative effects on the metabolic activity level (Figs. 4H and 6A).

Our results clearly showed donor-dependent effects on cytokine upregulation with (and without) drug treatment. The selective upregulation of the pro-inflammatory cytokines TNF-α and IL-16 in CoC1 derived from donor 10 after DIC treatment could explain the DILI effects in this donor. However, the concomitant upregulation of the anti-inflammatory cytokines IL-10, CCL22, and CCL23 by DIC treatment shows rather broad cytokine-induction mechanisms by DIC that are different from those induced by APAP. In general, the cells derived from donor 11 and especially from donor 12 were less responsive to DIC and APAP in terms of cytokine induction. These changes in cytokine expression match our observations on the metabolic activity results.

### The inflammatory phenotype of hepM and M0 macrophages in co-cultures remains unaltered following drug treatment

**Fig. 7:**
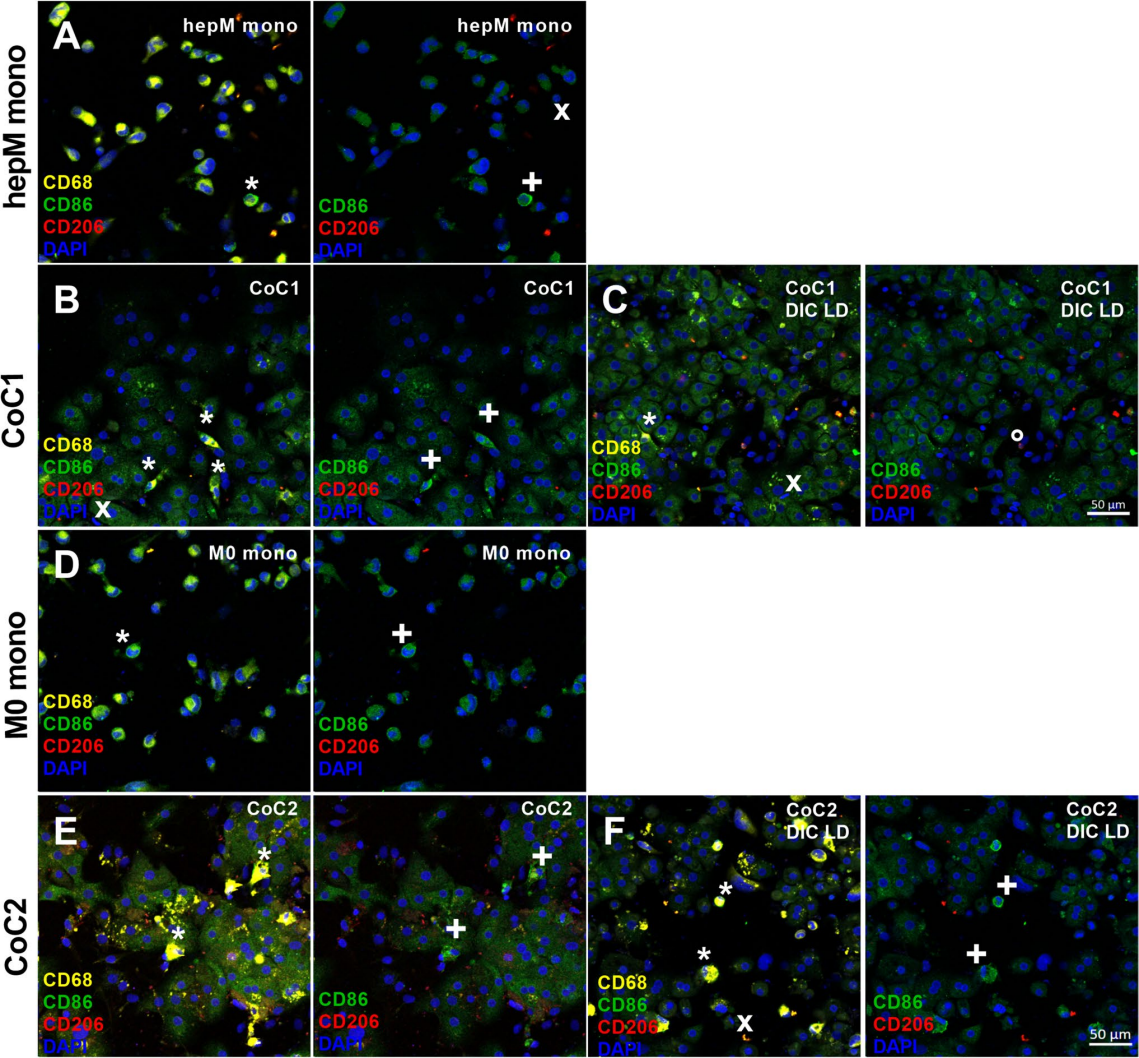
Immunofluorescence stainings of CD68, CD86 and CD206 to determine the inflammatory state of macrophages in the cultures. (**A**) HepM, (**B**) CoC1, and (**C**) CoC1 treated with low-dose diclofenac (DIC), and (**D**) M0, (**E**) CoC2, and (**F**) CoC2 treated with low-dose DIC were stained for the general macrophage marker CD68, the pro-inflammatory macrophage marker CD86, and the anti-inflammatory macrophage marker CD206. CD68^+^ macrophages (yellow) are marked with asterisks (*). CD86^+^ cells (green) are marked with a plus ( +). Rarely occurring CD206^+^ cells (red) are marked with a circle (°). Representative immunofluorescence images for donors 10, 11, and 12 are shown. CoC1 = PHH co-culture with hepM; CoC2 = PHH co-culture with M0 macrophages; mono = monoculture

In the next step, we analyzed the cell type–specific inflammatory phenotype of macrophages in the co-cultures before and after drug treatment. Specifically, we used immunofluorescence to evaluate the macrophage marker CD68, the pro-inflammatory macrophage marker CD86, and the anti-inflammatory marker CD206. We could clearly detect macrophages as CD68-positive cells (Fig. 7). After culturing for 36 h, the hepM and M0 monocultures were dominated by CD68^+^CD86^−^CD206^−^ macrophages, with a few CD68^+^CD86^+^ pro-inflammatory macrophages (Fig. 7A and D). The co-cultures showed the same macrophage populations, although with a much lower number of macrophages in general. Rarely, we observed anti-inflammatory CD68^+^CD206^+^ macrophages. Following drug treatment, there was a similar number of CD86^+^ hepM and CD206^+^ hepM compared with the vehicle control (Fig. 7C and F). Consequently, there was no qualitative difference between the control and treated cells in terms of the inflammatory phenotype.

## Discussion

In this study, we established an autologous co-culture model consisting of PHH and hepM, which represent the two major hepatic cell populations. To the best of our knowledge, for the first time we have cultured autologous PHH and human hepM in their natural ratio, mimicking the organ-specific immunological environment in vitro. We compared this co-culture model consisting of primary cells to a co-culture model consisting of PHH + M0 macrophages differentiated from the THP1 cell line to compare donor-related and standardized immunological macrophage reactions. For functional validation, we investigated DILI caused by the drugs MEN, DIC, and APAP. Consistent with our previous study (Kegel et al. 2015), drug-related macrophage reactions occurred in a donor-dependent manner. In this regard, the autologous co-culture systems were more sensitive in displaying DILI effects mediated by DIC and APAP.

We grew PHH, corresponding to hepM, and M0 macrophages in mono- and co-cultures for up to 60 h and characterized the culture stability and cell–cell interactions. We chose a PHH-to-macrophage co-culture ratio of 4:1 because this resembles the ratio in vivo in healthy liver (Lopez et al. 2011). Other groups have co-cultured hepatocytes and macrophages at a ratio of 2:1, but this is more likely to represent an inflammatory liver condition (Rose et al. 2016). After 36 h of co-culture, we observed two macrophage populations. The majority of macrophages were stationary and had a round morphology and highly active filopodia, suggesting phagocytic activity. In addition, we observed a migratory type that crawled under and between neighboring hepatocytes. It is generally accepted that due to their stationary behaviour, Kupffer cells are not optimally suited to migrate to injury sites (Ju and Tacke 2016). In contrast, monocyte-derived macrophages have been shown to migrate in high numbers to tissue injury (Zhao et al. 2024). Therefore, we conclude that the migratory macrophages we observed in our timelapse videos are monocyte-derived macrophages, and the stationary cells are Kupffer cells. This conclusion is further supported by the timelapse recordings of primary cell cultures derived from “pre-damaged” liver tissue after portal vein embolization, which display many highly active and migratory macrophages. In CoC2, we also observed the migratory, probably monocyte-derived macrophage type. However, this population was much less abundant and possibly due to contamination of the PHH batch, which is a known limitation of the liver cell isolation process (Godoy et al. 2016). The presence of various macrophage subpopulations explains the observed heterogeneity of the hepM monocultures. However, the expression of macrophage-specific markers also showed a certain degree of variability in the M0 monoculture.

Monitoring cell culture stability revealed slight signs of isolation stress in the PHH monocultures, measured as a time-dependent reduction in the protein content and initially elevated GGT activity. Hepatocyte death during the first 24 h of cell culture is quite common due to post-isolation stress (Damm et al. 2019). The co-culture with hepM showed elevated metabolic activity and reduced transaminase activity, indicating a beneficial effect on culture quality and potentially prolonging their longevity. However, this effect was accompanied by elevated LDH activity, suggesting macrophage stress. This high variability leads us to assume that this effect is batch dependent and therefore correlates with liver cell quality in general. The co-culture of PHH + M0 macrophages did not improve hepatocyte viability.

Injured hepatocytes secrete damage-associated molecular patterns (DAMPs) to activate Kupffer cells, which secrete chemokines/cytokines to attract and activate neutrophils (IL8 [CXCL8] and CXCL1 [GROα]), monocytes (CCL2 [MCP-1]), leukocytes (CCL5 [RANTES] and IL16), and stellate cells (TGF-β) (Zhao et al. 2024; Ju and Tacke 2016). Our characterization of the inflammatory state of the cell cultures showed that the PHH isolation stress was accompanied by secretion of CCL4 (MIP-1β) and CXCL1 (GROα). CCL4 is a chemokine that is produced during inflammation and tissue damage to attract cytotoxic immune cells such as natural killer (NK) and CD8^+^ cells (Ju and Tacke 2016). CCL4 is expressed in hepatocytes under cholestatic conditions in vitro (Allen et al. 2011) and secreted under hypoxic conditions in vitro (Vodovotz et al. 2019). CXCL1 is a DAMP and a chemoattractant for neutrophils. In the liver, hepatocytes are the main source of CXCL1 production in response to challenge from necrotic cells (Su et al. 2018), and neutrophils are responsible for clearance of damaged liver tissue (Reif et al. 2017). Both inflammatory cytokines were decreased in the co-cultures with hepM and M0, while the chemoattractants IL-8 and CXCL2 (GROβ) were increased. The CXCL2–IL-8 axis plays an important role in acute liver injury given the dependence of neutrophil and macrophage chemotaxis on CXCR1/CXCR2 ligands. Furthermore, this axis is of great interest due to the expression of CXCR1/CXCR2 by hepatocytes, enabling these chemokines to directly affect both acute inflammation and hepatocyte survival/function (Saiman and Friedman 2012). Indeed, NPC, which also include Kupffer cells, can support hepatocyte growth and survival in cell culture (Kaur et al. 2023). In addition, in the co-cultures with hepM, there were increased levels of IL-16, CCL23, and CCL24, which are effective chemoattractants for eosinophils. It was shown in multiple models of liver injury that the recruitment of eosinophiles had a protective role (Xu et al. 2022). In contrast, cells in CoC2 secrete macrophage migration inhibitory factor (MIF), which is a multi-potent cytokine that contributes to the inflammatory response to injury, as well as anti-inflammatory TGFβ, which activates hepatic stellate cells (Radosavljevic et al. 2024). We conclude that due to isolation and initial cultivation stress, our PHH cultures were in an inflammatory state. Co-cultivation with hepM and M0 macrophages reduced the initial inflammatory state and potentially activated survival and/or regenerative pathways. Of note, only CoC1 presented improved culture quality. Nonetheless, after 36 h of co-culture, CoC1 and CoC2 still showed signs of inflammation based on the presence of cytokines/chemokines that are linked to the recruitment and activation of other immune cells. Therefore, hepatic macrophages take on the position of central moderators in sensing hepatocyte stress and communicating it to other immune cells. Additionally, hepM are capable of improving hepatocyte culture quality and, potentially, longevity.

PHH monocultures have long been the gold standard for drug-related hepatotoxicity assays. However, they often do not mimic hepatotoxicity sufficiently. As shown in mouse studies, NPC can aggravate or mitigate the hepatotoxicity of drugs (Tasnim et al. 2021). We assessed the potential application of our established autologous PHH + hepM co-culture system by examining whether treatment with subtoxic concentrations of known hepatotoxic compounds—MEN, DIC, and APAP—led to additional inflammatory reactions. Each compound reduced metabolic activity in cultures of cells derived from donor 10 or 11, but not with cells derived from donor 12. Donors 10 and 11 were patients with liver metastasis without underlying liver disease, although they had received chemotherapy. Donor 12 was a patient with an intrahepatic cholangiocarcinoma, which usually develops on the background of chronic liver disease. Our group recently reported that most Kupffer cells from healthy liver tissue show an anti-inflammatory phenotype (Zimmermann et al. 2021). Here, our characterization data revealed that the PHH + hepM co-cultures present inflammation due to isolation and initial cultivation stress. Additional cell stress from drug treatment should lead to further inflammation. We confirmed this phenomenon with immunofluorescence of treated and untreated co-cultures, both of which showed a rather pro-inflammatory macrophage population. Furthermore, drug treatment led to reduction in metabolic activity, suggesting a decrease in hepatocyte viability in mono- and co-cultures of cells derived from donor 10 or 11, but not with cells derived from donor 12. This correlates with results from our previous study, where we reported that hepM isolated from chronically damaged tissue and treated with supernatants from drug-treated PHH showed dominant anti-inflammatory reactions (Kegel et al. 2015). Based on these data, we hypothesize that cells derived from donor 12 originate from chronically damaged liver tissue and contain hepatocytes with high potential for survival. Consequently, there would be no further benefit from additional co-cultivation with hepM. Notably, we also observed an increased metabolic activity in CoC2 of donor 12 upon high dose MEN treatment. We interpreted this phenomenon as a mutual activation of M0 and PHH that leads to MEN resistance. Similar effects were described by Timblin et al. (Timblin et al. 2021), although not in a co-culture system, but after co-treatment of macrophages with mitochondrial stress triggers. The same effect was seen for CoC1 of donor 11 after high dose APAP treatment. In summary, the PHH + M0 co-cultures showed either no or milder reactions to drug treatment compared with the PHH + hepM co-cultures. We have previously shown that chronic tissue damage is associated with a shift to an increasing number of monocyte-derived macrophages with a dominant anti-inflammatory phenotype (Zimmermann et al. 2021). Therefore, M0, which are THP1 derived, might react similarly to monocyte derived macrophages when co-cultured with PHH.

Our cytokine and chemokine data related to co-cultures of cells derived from donor 10 link reduced metabolic activity to immune cell activation. Interestingly, CoC1 with cells derived from donor 10 showed increased TNF-α when treated with DIC compared with the PHH monoculture, while TNF-α was not induced by DIC in the hepM monoculture. This suggests that TNF-α could be a mediator of toxicity induced by DIC. TNF-α is known to either promote hepatocyte growth or death; it is suggested to act in a concentration-dependent manner via yes associated protein (YAP) signaling, with high TNF-α concentrations blocking YAP resulting in cell death (Shuh et al. 2013; Zhao et al. 2020). Consistently, DIC enhanced cytotoxicity when co-incubated with TNF-α in a simple HepG2 monoculture, indicating its relevance as an initiator of idiosyncratic DILI. Additionally, DIC showed enhanced cytotoxicity in co-cultures of HepG2 and proinflammatory M0 (lipopolysaccharide-stimulated THP1 derived macrophages) (Granitzny et al. 2017). In our initial characterization, we observed the presence of chemoattractants for eosinophils (IL-16 and CCL23) and macrophages (CCL22) in the hepM and partly M0 co-cultures, respectively. These cytokines were further increased after DIC treatment of CoC1 with cells derived from donor 10, which indicates a general macrophage reaction toward hepatocyte stress. In addition, other cytokines, including pro-inflammatory IL-6 and anti-inflammatory IL-10, were upregulated in CoC1 with cells derived from donor 10 but not with cells derived from donor 11 or 12, indicating the sensitivity of cells derived from donor 10 to DILI. In contrast, the co-cultures with cells derived from donor 11 did not show impaired metabolic activity after DIC treatment, but its PHH monoculture did. Cytokine analysis confirmed that there was no further inflammatory reaction in the co-cultures. However, for donor 11, there was elevated GGT release in the PHH monoculture and CoC2 but not CoC1, suggesting that the DILI-causing drugs had a reduced toxic impact.

Taken together, we observed donor- and drug-dependent DILI effects when treating co-cultures of hepM and M0 with DIC or APAP. Despite marked variability in the results, our results indicate that hepM co-cultures are the more appropriate and more sensitive test system to detect DILI effects. Notably, PHH isolated from chronically damaged tissues were less suitable for toxicity studies. Further, hepM isolated from chronically damaged tissues as well as M0 were less responsive and thus less capable of mimicking hepatotoxicity.

Since the initial reports from (Wewering et al. 2017), presenting clear evidence that hepatocyte–immune cell co-cultures represent an improved system to evaluate hepatotoxicity, there have been great advances to develop human-based in vitro systems to assess DILI. Although newer systems have used primary cells and have improved cultivation techniques, other systems are either not autologous or still include cell lines (Bronsard et al. 2024; Shinozawa et al. 2021; Padberg et al. 2021; Granitzny et al. 2017). Due to limited accessibility to hepatocytes and hepatic immune cells, it is challenging to frequently obtain cells from the same donor, leading to limited possibilities of obtaining human leukocyte antigen (HLA)-matched co-cultures. This might be a problem given that HLA-unmatched co-cultures have been shown to activate Kupffer and T cells (Tasnim et al. 2021). In the present study, we addressed this important limitation of other co-culture systems. However, our system still has the known limitations of primary cell applications in terms of donor dependence and a lack of reproducibility. The number of isolated hepM was the bottleneck for the number of experiments that we could perform in parallel. In addition, M0 differentiation of THP1 cells has to be successful prior to each primary cell isolation to gain full batches of all cells for the study design. Only a low number of donors provide the sufficient number of cells required for the extensive study design detailed in Fig. 1, and failures in the M0 differentiation further limited their number. Therefore, our study was limited by the use of only three donors. We conclude that macrophage-related immunological reactions are donor specific and that the complex in vitro model consisting of autologous primary human cells is influenced by many patient-related factors. Therefore, the results have to be considered on a case-by-case basis until additional donors are investigated to validate these data. Restriction to a co-culture using hepM and reduction of the read-outs should allow the investigation of DILI in a broader patient cohort. Additional improvements of the study design should include a switch from a two-dimensional (2D) culture to a three-dimensional (3D) culture system like a PHH sandwich culture with immune cells. We have already shown that 3D sandwich cultures of PHH show reduced culture stress due to more physiological cell–cell contacts between adjacent hepatocytes (Deharde et al. 2016), as well as a more in vivo-like xenobiotic metabolism (Schyschka et al. 2013). In this regard, the xenobiotic metabolism of test compounds should be investigated by liquid chromatography–mass spectrometry (LC–MS) analyses to verify the production of active metabolites. A disadvantage of 3D cultures is that dead cells cannot be removed because they are trapped between the extracellular matrix layers. 3D co-cultures could solve this problem due to the ability of macrophages to eliminate dead cells. To increase the sensitivity of detecting DILI effects, it would be of interest to include additional immune cells in the primary PHH + hepM co-culture system. (Roser et al. 2023) co-cultured HepaRG cells or PHH with CD8^+^ T cells or NK cells and monocyte-derived macrophages and showed that hepatocytes with CD8^+^ T cells and monocyte-derived macrophages display aldesleukin-mediated cytotoxicity. However, co-cultures with PHH revealed high donor variability in DILI results, demonstrating the difficulties in generating primary hepatocyte culture systems to establish reliable DILI in vitro cultures (Roser et al. 2023). However, the donor variabilities in models consisting of primary human cells (e.g. CoC1) reflect the situation therapists face in everyday patient care. Humans are individuals and thus modern medicine turns towards personalized diagnostics and therapies. Therefore, models like our autologous primary cell co-culture system, which reflect donor-specific drug responses, may provide opportunities as future test systems to identify a high risk for hepatotoxicity in patients.

Taken together, we have presented here for the first time an autologous co-culture system consisting of PHH + hepM. It reproduces DILI caused by MEN, DIC, or APAP in a donor-dependent manner. HepM can either promote or reduce the DILI effects in these co-culture systems. The co-cultures of PHH + M0 macrophages derived from THP1 cells are slightly less sensitive than co-cultures with PHH + hepM in representing DILI. Therefore, we recommend using PHH + hepM for DILI studies. In the future, additional DILI parameters such as the detection of toxic metabolites by LC–MS or the evaluation of cytotoxicity by cytotoxic immune cells could be included in the evaluation of DILI to expand the spectrum of observable effects.

## Materials and methods

### Liver samples derived from patients undergoing liver resection

Liver tissue from liver resections was obtained from Leipzig University Hospital, Department of Hepatobiliary Surgery and Visceral Transplantation. During liver resections, diseased tissue and the surrounding unaffected liver tissue were removed; a sample of the unaffected liver tissue was used for cell isolation. The patients provided written informed consent in accordance with the approved ethical protocols. Liver samples from 11 patients were included in this study. The donor characteristics are displayed in Table 1.

### Cell isolation

PHH and hepM were isolated from the same liver tissue samples with a two-step collagenase perfusion technique described previously (Kegel et al. 2016; Damm et al. 2019). Briefly, liver tissue was perfused with a perfusion buffer containing EGTA (cat. no. 03777-10 g, Sigma-Aldrich, St. Louis, USA) followed by a digestion buffer containing collagenase P (cat. no. 11213873001, Roche, Basel, Switzerland). PHH were obtained by washing and centrifuging at 51 x*g* the generated cell suspension two times with phosphate-buffered saline (PBS) containing Mg^2+^/Ca^2+^ (cat. no. 14040174, Gibco, Thermo Fisher Scientific, Waltham, Massachusetts, USA). The PHH in the resulting pellet were resuspended and for PHH monoculture (see section Co-cultures and monocultures) seeded on cell culture plates coated with rat tail collagen I (produced in-house); the supernatant containing hepM and other non-parenchymal cells was centrifuged at 300 x*g* and then 600 x*g*. The resulting cell pellets containing hepM were combined. HepM were separated from the other non-parenchymal cells by adhesion separation. For this, the cells were seeded for 20 min on rat tail collagen I–coated cell culture plates. After 20 min, the hepM had attached to the cell cultures plate, and the non-adherent cells were removed resulting in hepM monoculture (see section Co-cultures and monocultures).

### Cell culture

#### THP-1 culture and differentiation into M0 macrophages

THP-1 cells were cultured in suspension in RPMI 1640 medium (Sigma-Aldrich, St. Louis, USA) containing fetal calf serum (FCS; cat. no. S0615-500 ml Sigma-Aldrich, St. Louis, USA), penicillin/streptomycin (cat. no. 15140122, Gibco, Thermo Fisher Scientific, Waltham, Massachusetts, USA) and L-Glutamine 200 mM (cat. no. 25030–024, Gibco, Thermo Fisher Scientific, Waltham, Massachusetts, USA) maintaining a density of 0.1–1 million cells/mL. The culture medium was refreshed every 2–3 days. Cells were collected by centrifugation at 300xg for 5 min, washed in PBS to remove debris, and resuspended in fresh medium. Cell viability and count were assessed prior to reseeding into new culture flasks.

For M0 macrophage differentiation, THP-1 cells were seeded on rat tail collagen I-coated cell culture plates in medium containing 10 ng/mL phorbol 12-myristate 13-acetate (PMA, P8139-1MG, Sigma-Aldrich, St. Louis, USA). After 24 h of incubation, adherent cells were washed twice with 400 µL PBS resulting in M0 monocultures.

#### Co*-*cultures and monocultures

For co-cultures PHH were seeded in a ratio of 4:1 on the respective macrophage monoculture and for the PHH monoculture the respective share of PHH were seeded in Williams E medium with GlutaMAX^™^ (cat. no. 32551087, Gibco, Thermo Fisher Scientific, Waltham, Massachusetts, USA) with fetal calf serum (FCS; cat. no. S0615-500 ml Sigma-Aldrich, St. Louis, USA), insulin (cat. no. PZN: 02526396, Lilly, Germany), dexamethasone (cat. no. PZN: 08704404, Jenapharm/MIBE GmbH, Germany), HEPES (cat. no. 15630–056, Gibco, Thermo Fisher Scientific, Waltham, Massachusetts, USA), sodium pyruvate (cat. no. 11360–039, Gibco, Thermo Fisher Scientific, Waltham, Massachusetts, USA), minimum essential medium non-essential amino acids (MEM-NEAA; cat. no. 11140–035, Gibco, Thermo Fisher Scientific, Waltham, Massachusetts, USA), and penicillin/streptomycin (cat. no. 15140122, Gibco, Thermo Fisher Scientific, Waltham, Massachusetts, USA). In parallel the medium of the macrophage monocultures were changed to the same medium mentioned before. The PHH were allowed to adhere overnight (12–16 h). Some analyses used supernatant directly after the isolation. The later was obtained 2 h after seeding (shortest sampling time point after cell adherence = 0 h of cultivation). The next day, the cells were washed two times with PBS containing Mg^2+^/Ca^2+^ to remove dead cells, FCS, insulin, and dexamethasone (sample = initial 12 h of cultivation). Thereafter, the cells were cultured in Williams E medium with GlutaMAX™ with HEPES, sodium pyruvate, MEM-NEAA, and penicillin/streptomycin, but without FCS, insulin, and dexamethasone (starvation medium) for another 24 h. After 24 h culturing in starving medium (sample = initial 36 h of cultivation), the initial characterization was performed (see Fig. 1). Then, the cultures were treated with a low or high dose of MEN (1 and 10 µM; cat. no. M5625-25G, Sigma-Aldrich, St. Louis, USA), DIC (0.5 and 5 mM; cat. no. D6899-10G, Sigma-Aldrich, St. Louis, USA), or APAP (0.5 and 5 Mm; cat. no. A5000-100G, Sigma-Aldrich, St. Louis, USA). MEN, DIC and APAP were dissolved in dimethyl sulfoxide (DMSO). The doses and incubation time were selected according to pretests (Fig. S1). After the treatment (3 h for MEN, and 6 h DIC and APAP), the DILI characterization was performed with the assays outlined in Fig. 1.

### XTT assay

The Cell Proliferation Kit II (XTT) Kit (cat. no.11465015001, Roche, Switzerland) was used to measure metabolic activity according to the manufacturer’s instructions. Briefly, the culture medium was aspirated from the cells and the cells were washed once with PBS. XTT labeling solution was added to the cells and incubated for 2 h at 37°C and 5% CO_2_. The optical density (OD) was determined at 492 and 690 nm with a microplate reader (Synergy H1, BioTek Instruments, Inc., Winooski, VT, USA). For analysis, the OD reading at 690 nm was subtracted from the OD reading at 492 nm.

### 2′,7′-dichlorofluorescein (DCF) assay

To quantify intracellular ROS levels in the cultures 2′,7′-dichlorofluorescein (DCF) assay was performed. The assay uses 2′,7′-dichlorofluorescein diacetate (DCF-DA), a non-fluorescent compound that diffuses into cells. Intracellular esterases convert it into 2′,7′-dichlorodihydrofluorescein, which is then oxidized by ROS to form fluorescent 2′,7′-dichlorofluorescein (DCF). The fluorescence intensity, measured with a fluorometric plate reader, reflects the intracellular ROS levels.

Cells were washed with PBS and incubated with 20 µM DCF-DA (sc-209391, Santa Cruz Biotechnology, Dallas, USA) diluted in serum-free media for 30 min at 37°C, 5% CO_2_. DCF-DA was removed from the cells and replaced by serum-free media. Cells were incubated for 1 h at 37°C, 5% CO_2_. The supernatant was transferred to a 96-well plate and fluorescence intensity was measured with excitation at 480 nm and emission at 530 nm using a microplate reader.

### Bicinchoninic acid (BCA) protein assay

The protein content of cultures was assessed for normalization purposes using the BCA protein assay. Briefly, cultures were washed twice with PBS containing Mg^2+/^Ca^2+^ and then lysed using a BCA lysis buffer containing 0.1% sodium dodecyl sulfate (SDS), 0.5% Triton X-100, and 50 mM Trizma base, followed by ultrasonication. Samples were incubated with a BCA reaction mixture (cat. no. B9643-1L (BCA reagent A) and cat. no. C2284-25ML-D (CuSO4 Solution 4%), Sigma-Aldrich, St. Louis, USA) and bovine serum albumin (BSA, cat. no. A4503, Sigma-Aldrich, St. Louis, USA) used to prepare a standard curve according to the manufacturer’s instructions. The samples were incubated for 30 min at 37°C in the dark and the absorbance at 550 nm was measured using a microplate reader.

### ALAT, ASAT, GGT, and LDH activity assay

ALAT, ASAT, GGT and LDH are enzymes released upon cell membrane damage indicating cell death. The activity of these liver enzymes was assessed in the culture supernatants to determine occurrence of cell death and hepatotoxicity of substances. Culture supernatants were analyzed with commercially available reagents and kits (Diacon N [multi-color serum, normal], cat. no. D14481; Diacal Auto [multi calibration serum], cat. no. D98485; GGT, cat. no. D95604; LDH, cat.no. D95604; GOT [ASAT], cat. no. D95604; GPT [ALAT], cat. no. D95604, all from DIALAB, Wiener Neudorf, Austria) according to the manufacturer’s instructions. Absorbance was measured at 340 nm for ALAT, ASAT, and LDH, and at 405 nm for GGT with a microplate reader.

### Cytokine array

Conditioned media from PHH, hepM, M0, CoC1, and CoC2 (derived from donors 10, 11, and 12) were pooled to determine the cytokine profile using the RayBio^®^ Human Cytokine Array C5 (BioCat, Heidelberg, Germany) in triplicate. The arrays were performed according to the manufacturer’s instructions, with overnight incubation of the samples at 4 °C. Chemiluminescent signals were detected with a CCD camera (INTAS, Göttingen, Germany) and quantified with ImageJ v1.47 (National Institutes of Health, Bethesda, MD, USA). After background correction (blank values), all spots from one membrane were normalized to the respective positive controls. Targets with signal intensities below the standard error of the mean of the six positive controls (detection limit) were excluded from further analyses. Relative changes in the cytokine levels are presented as a heat map.

### ELISA

ELISA were performed on cell culture supernatants of PHH, CoC1 and CoC2 cultures with and without DIC and APAP low dose treatment. The following kits were used: human anti-TNFα ELISA (cat. no. 900-TM2, PeproTech, Cranbury, USA), human anti-IL1b ELISA (cat. no. 900-M95, PeproTech, Cranbury, USA), human anti-IL6 ELISA (cat. no. 900-M16, PeproTech, Cranbury, USA), human anti-IL10 ELISA (cat. no. 900-M21, PeproTech, Cranbury, USA), human anti-CCL22 DuoSet ELISA (cat. no. DY336, R&D Systems, Minneapolis, USA), human anti-CCL23 DuoSet ELISA (cat. no. DY131, R&D Systems, Minneapolis, USA), human TGF-beta 1 DuoSet ELISA (cat. no. DY240, R&D Systems, Minneapolis, USA), and human anti IL-16 ELISA (cat. no. D1600, R&D Systems, Minneapolis, USA). The ELISAs were performed according to the manufacturers’ instructions. A second-order polynomial (quadratic) standard curve was used for analysis.

#### Immunofluorescence

CD68, CD86, and CD206 immunofluorescence was performed according to a standard protocol. Briefly, the cultures were fixed for 15 min at room temperature, followed by permeabilization with 0.2% Triton X-100 for 10 min. The cells were incubated with 2% BSA for 20 min, and FcR was blocked by an FcR blocking reagent (cat. no. 130–059–901, Miltenyi Biotec, Bergisch Gladbach, Germany) for 10 min at 4°C. The cells were incubated with the appropriate primary antibody overnight at 4°C: mouse anti-CD68 (cat. no. ab955, Abcam, Cambridge, United Kingdom), rabbit anti-CD86 (cat. no. ab239075, Abcam, Cambridge, United Kingdom), and goat anti-CD206 (cat.no. sc34577, Santa Cruz Biotechnology, Dallas, Texas, USA). Subsequently, the cells were incubated with the appropriate secondary antibody for 1 h at room temperature: donkey anti-mouse Dylight 550 (cat.no. ab98767, Abcam, Cambridge, United Kingdom), donkey anti-rabbit Alexa Fluor 488 (cat.no. ab181346, Abcam, Cambridge, United Kingdom), and donkey anti-goat Alexa Flour 647 (cat. no. 705-605-147, Jackson ImmunoResearch, Ely, United Kingdom). Nuclear counterstaining was performed by Hoechst 33,342 for 10 min at room temperature. Mowiol (cat.no. 0713, Carl Roth, Karlsruhe, Germany) generated according to manufacturer’s instructions was used for mounting. Images were taken with a Keyence microscope BZ-9000 (Keyence GmbH, Neu-Isenburg, Germany).

#### Western blotting

The protein concentrations of the samples were determined by using the BCA Protein Assay (Thermo Fisher Scientific, Waltham, Massachusetts, USA). 25 µg of each sample were separated by SDS–polyacrylamide gel electrophoresis (PAGE). The separated protein was transferred onto a 0.2 µm nitrocellulose membrane by electroblotting. The membrane was incubated with 5% milk in Tris-buffered saline containing 0.1% Tween 20 (TBS-T) for 60 min, prior to incubating with the appropriate primary antibody diluted in milk overnight at 4 °C: PARP1 (1:2000; cat. no. 9542, Cell Signaling, Danvers, Massachusetts, USA) and beta-actin (1:5000; cat. no. 4970, Cell Signaling, Danvers, Massachusetts, USA). The next day, the blots were washed three times in TBS-T, incubated with the appropriate horseradish peroxidase-conjugated secondary antibody (1:5000) for 60 min, and then washed three times with TBS-T. Finally, protein bands were visualized by chemiluminescence using the SuperSignal^™^ West Femto Maximum Sensitivity Substrate (Thermo Fisher Scientific, Waltham, Massachusetts, USA) and scanned with the ImageQuant LAS-4000 chemiluminescence detection system (GE Healthcare, Chicago, Illinois, USA). A second way to normalize the Western blot data was the Revert^™^ 700 Total Protein Stain (LI-COR Biosciences, Bad Homburg, Germany). It is a non-permanent staining procedure to measure the total amount of protein in the different samples. Briefly, after transfer of the proteins, the membrane was rinsed with water and incubated in Revert 700 Total Protein Stain. After one wash step with the Wash solution, the total protein stain was acquired by using the Odyssey Imaging System. The staining was removed by using the Revert Destaining Solution and the membrane was used for further Western blot analysis, as described above.

#### Graphical design and statistical analysis

Experiments were performed with three biological replicates (three donors per experiment) except for evaluating the drug treatment times and concentrations and Western blotting, which was only performed for two donors. The data in Figs. 2 and 7 are presented as the means of at least three biological replicates + standard deviations (SD). The data in Figs. 3 and 5 are presented as the mean of three technical replicates ± SD. Figure 4 presents data of three pooled donors. GraphPad Prism 7 software (GraphPad Software, San Diego, CA, USA) was used for statistical analyses and to generate the graphs. To compare multiple groups, one-way and two-way analysis of variance (ANOVA) were performed followed by the post hoc Tukey test. Statistical significance is denoted in the figures with asterisks: * p < 0.05, ** p < 0.01, *** p < 0.001, and **** p < 0.0001. Servier Medical Art was used to generate the experimental design scheme in Fig. 1.

## Supplementary Information

Below is the link to the electronic supplementary material.

Supplementary file1 (DOCX 3304 KB)

Supplementary file2 (AVI 344261 KB)

Supplementary file3 (AVI 75754 KB)

Supplementary file4 (AVI 95140 KB)

Supplementary file5 (AVI 635890 KB)

Supplementary file6 (AVI 6430 KB)

Supplementary file7 (AVI 107782 KB)

Supplementary file8 (AVI 92729 KB)

## Declarations

### Data availability

The data presented in this study are available on request from the corresponding author. The data are not publicly available due to data protection regulations.
