# Supplementary material for: A new human autologous hepatocyte/macrophage co-culture system that mimics drug-induced liver injury–like inflammation
Source: Arch Toxicol. 2024 Dec 22;99(3):1167–85. doi: 10.1007/s00204-024-03943-8 (PMC11821741; doi:10.1007/s00204-024-03943-8)
Supplement: Supplementary file 1 — Supplementary file1 (DOCX 3304 KB) [file 204_2024_3943_MOESM1_ESM.docx]

**Supplementary Figures**

*A new human autologous hepatocyte/macrophage co-culture system that mimics drug-induced liver injury–like inflammation*

Andrea Zimmermann^1,2, https://orcid.org/0000-0001-6603-8704*^, Andrea Scheffschick^1,2, https://orcid.org/0000-0002-2848-2617*^, René Hänsel^1,3, https://orcid.org/0000-0001-8344-0658^, Hannes Borchardt^4^, Jia Li Liu^5^, Sabrina Ehnert^6, https://orcid.org/0000-0003-4347-1702^, Gerda Schicht^1,2, https://orcid.org/0000-0002-2799-6210^, Lena Seidemann^1, https://orcid.org/0000-0002-1531-7735^, Achim Aigner^4, https://orcid.org/0000-0002-2778-6256^, Susanne Schiffmann^7, https://orcid.org/0000-0001-5035-2504^, Andreas Nüssler^6, https://orcid.org/0000-0002-6666-6791^, Daniel Seehofer^1,2,5, https://orcid.org/0000-0002-7492-8309^, Georg Damm^1,2,5, https://orcid.org/0000-0002-2104-8076^

^1^ Department of Hepatobiliary Surgery and Visceral Transplantation, Clinic and Polyclinic for Visceral, Transplant, Thoracic and Vascular Surgery, Leipzig University Medical Center, Leipzig, Germany

^2^ Saxonian Incubator for Clinical Translation (SIKT), University of Leipzig, Leipzig, Germany

^3^ Institute for Medical Informatics, Statistics and Epidemiology (IMISE), Leipzig University, Leipzig, Germany

^4^ Rudolf-Boehm-Institute for Pharmacology and Toxicology, Clinical Pharmacology, Faculty of Medicine, University of Leipzig, Leipzig, Germany

^5^ Department of General-, Visceral- and Transplantation Surgery, Charité - University Medicine Berlin, Berlin, Germany

^6^ Department of Traumatology, BG Trauma center, University of Tübingen, Tübingen, Germany

^7^ Fraunhofer Institute for Translational Medicine and Pharmacology ITMP, Frankfurt am Main, Germany

* Andrea Zimmermann and Andrea Scheffschick have contributed equally to the study and the generation of the manuscript

Corresponding Author: Dr. rer. nat. Georg Damm, [georg.damm@medizin.uni‑leipzig.de](mailto:georg.damm@medizin.unileipzig.de), https://orcid.org/0000-0002-2104-8076


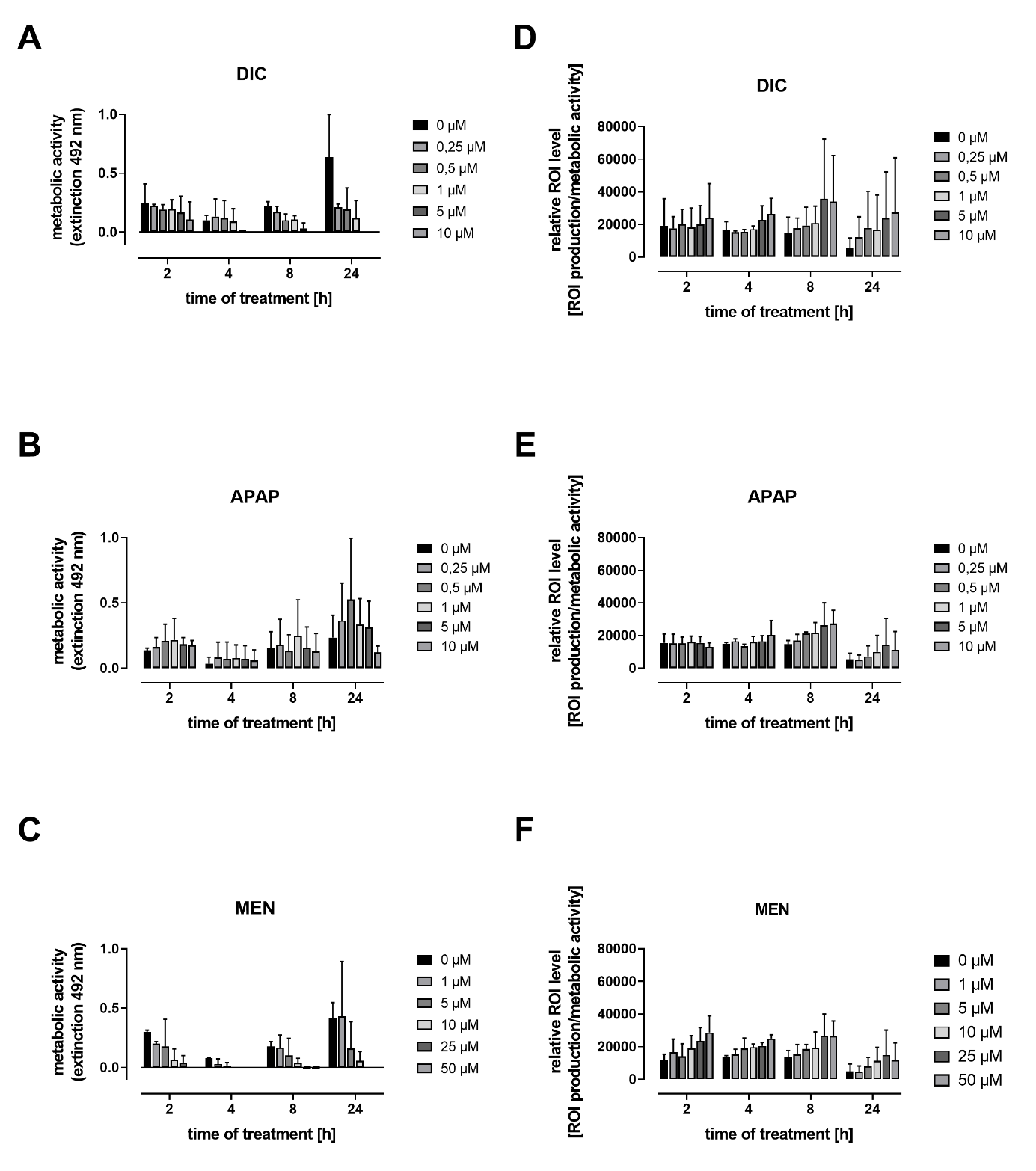


**Fig. S1 Testing of concentration and time points for DIC, APAP and MEN treatment on PHH**. PHH have been treated for 2-24 h with different concentrations of (**A, D**) DIC, (**B, E**) APAP or (**C, F**) MEN followed by analysis of metabolic activity using XTT assay and ROI production using DCF assay. A reduction of metabolic activity and ROI increase following substance treatment compared to the respective time control was considered as cytotoxic. Data are presented as two biological replicates of donor 07 and 08


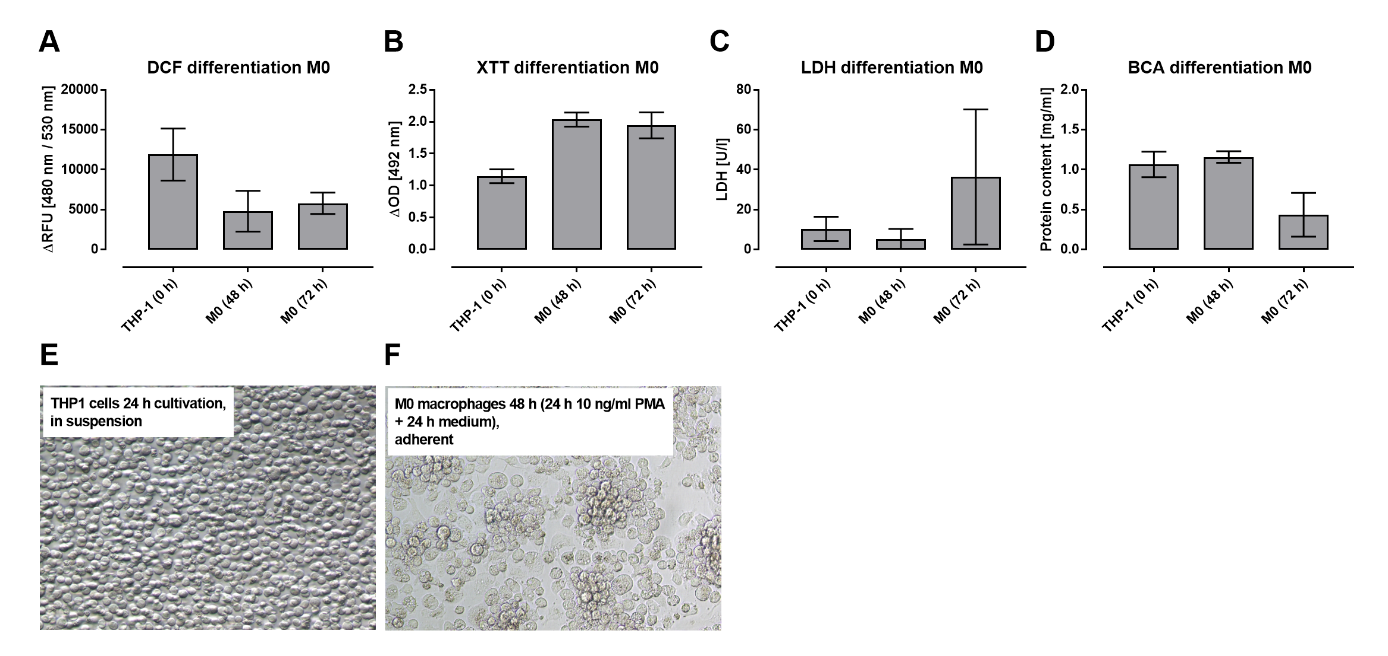


**Fig. S2 Differentiation of THP1 cells to M0 macrophages.** THP1 cells have been treated with 10 ng/ml phorbol-12-myristate-13-acetate (PMA) for 24 h followed by cultivation for an additional 24 h or 48 h in medium without PMA. (**A**) ROI formation (DCF assay), (**B**) metabolic activity (XTT assay), (**C**) LDH release and (**D**) protein content (BCA assay) were monitored up to 72 h to assess differentiation

**
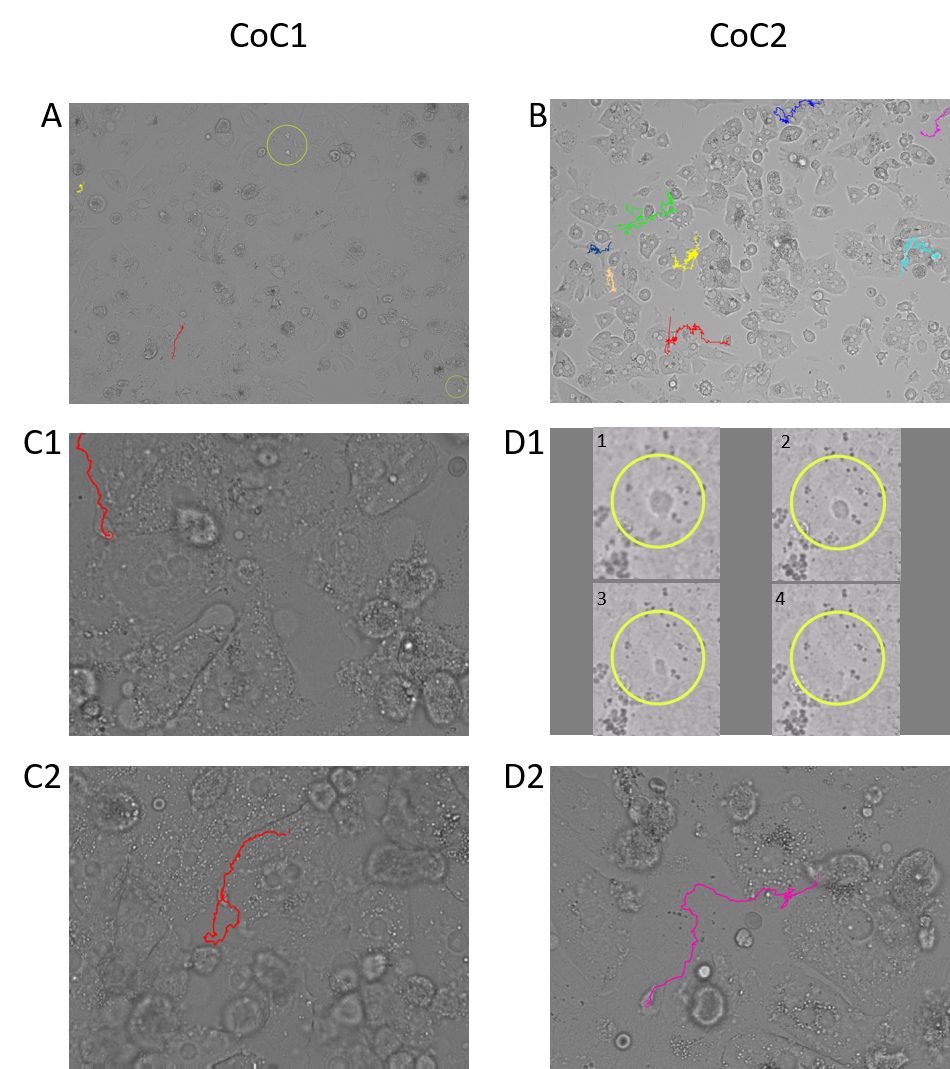
**

**Fig. S3 Macrophage dynamic and behavior in co-cultures of PHH + hepM as well as PHH + M0.** Dynamic of macrophages is shown as macrophage tracking here shown as representative screenshots of time laps recordings of (**A**) co-culture of PHH + hepM of D13 (CoC1, see Suppl File 2) and (**B**) co-culture of PHH + M0 macrophages of D04 (CoC2, see Suppl. File 3) after 36 h of cultivation. Macrophage behavior is shown in representative region of interest screenshots for CoC1 of D14 showing (**C1**) spheric shaped hepM presenting filopodia (see Suppl. File 4), (**C2**) elongated hepM crawling between adjacent PHH (Suppl. File 5) and for CoC2 of D14 and D15 showing (**D1**) a transition from a spheric in an elongated M0 (see Suppl. File 6) and (**D2**) elongated M0 crawling under and between adjacent PHH (see Suppl. File 7)


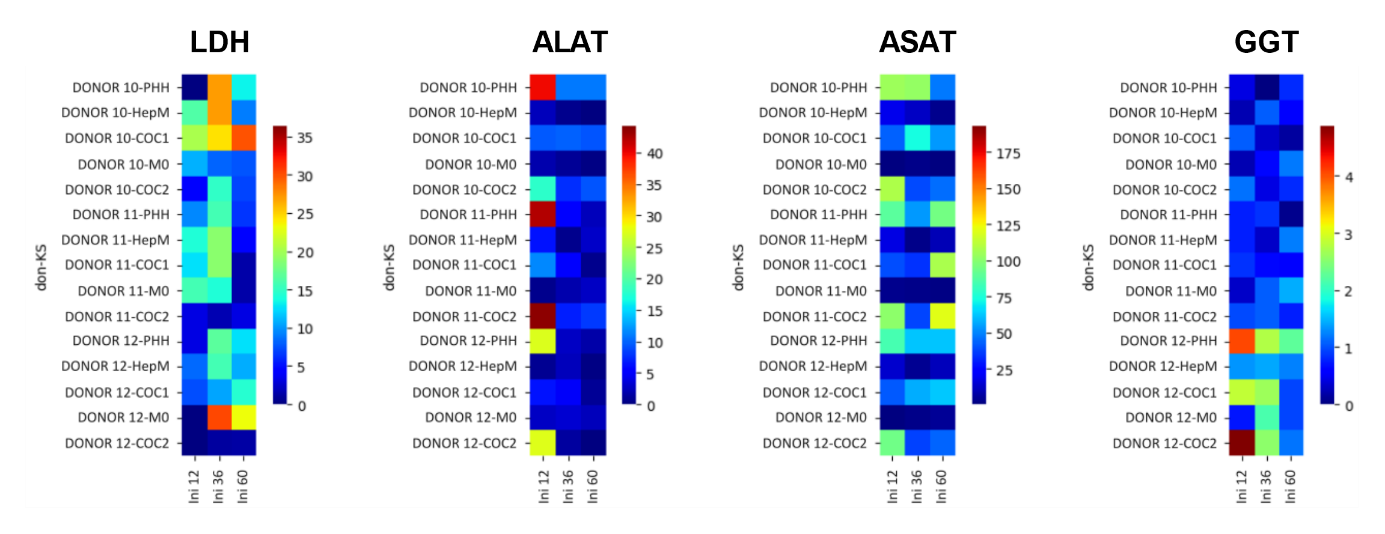


**Fig. S4 Analysis of LDH, ALAT, ASAT, and GGT in PHH and macrophage mono- and co-cultures after 12 h, 36 h and 60 h of cell culture.** Heat map shows the release of the enzymes LDH, ALAT, ASAT, and GGT in the cell culture supernatants of the mono- and co-cultures. Blue indicates low activity of the enzymes. Red indicates high activity of the enzymes


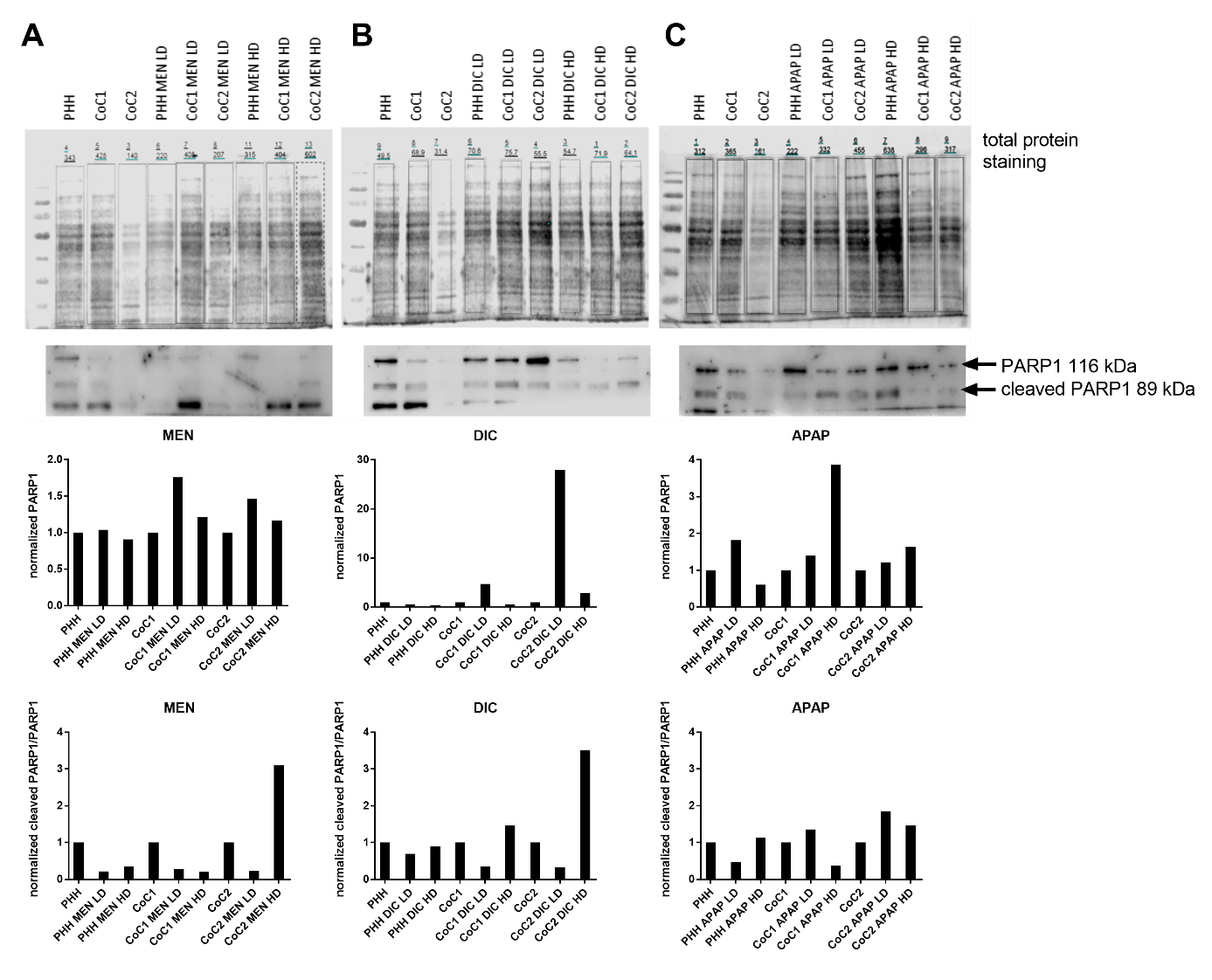


**Fig. S5 Western blotting analysis of PARP1 in mono- and co-cultures following MEN, DIC and APAP treatment.** After 36 h of cultivation, the cultures were treated with low (LD) or high (HD) dose (**A**) for 3 h with MEN, (**B**) for 6 h with DIC or (**C**) APAP. Western blots were performed for PARP1 (116 kDa) and total protein staining for normalization. Additional bands at 89 kDa correspond to cleaved PARP1. The presented Western blots are representative blots of Donor 11. Bar graphs represent the ratios of cleaved PARP/PARP


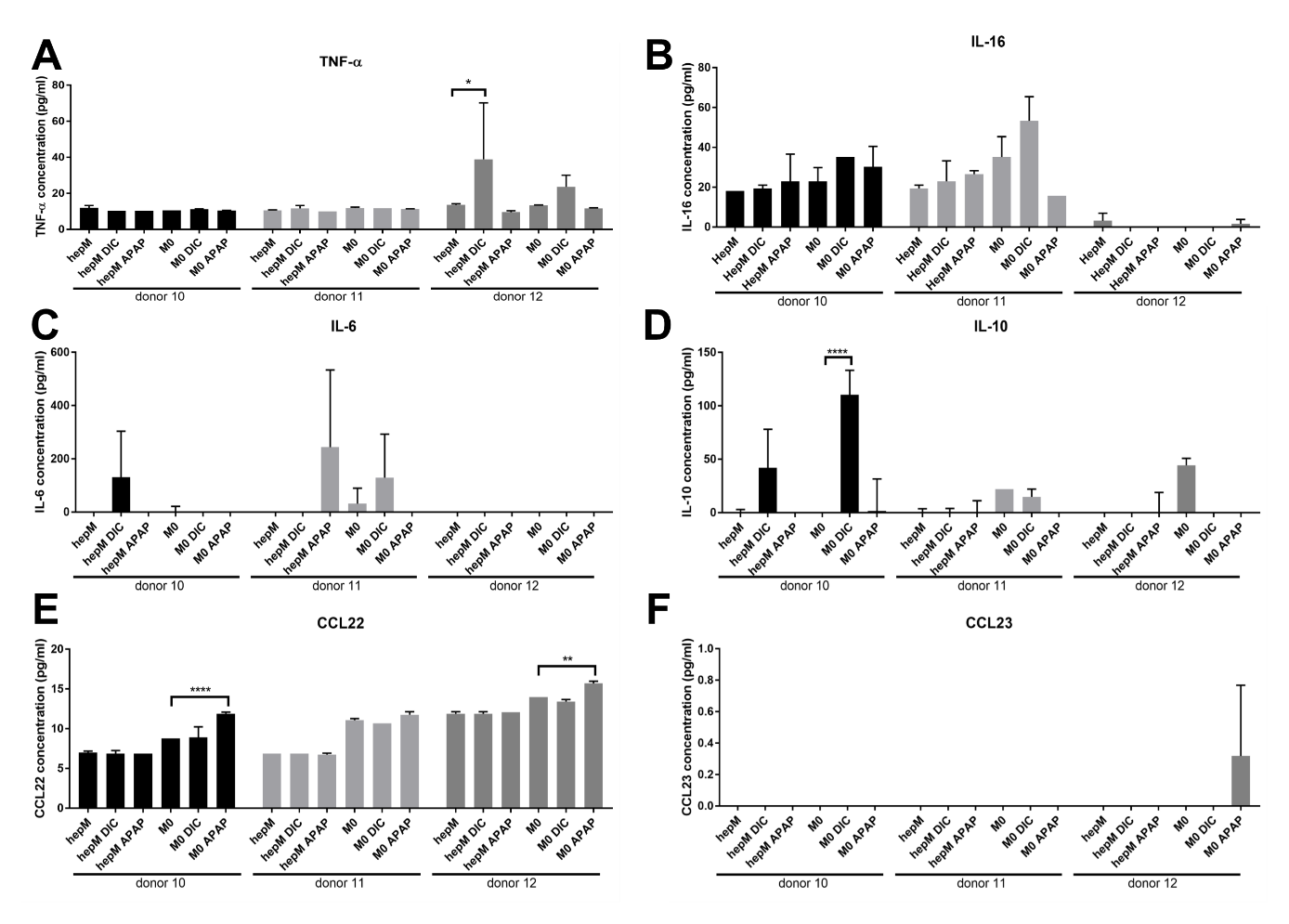


**Fig. S6 Analysis of cytokines and chemokines in macrophage monocultures following DIC and APAP treatment.** ELISA analysis of TNF-α, IL-16, IL-6, IL-10, CCL22 and CCL23 after low dose DIC and APAP treatment is shown in (**A-F**). Data for hepM is shown as three pooled technical triplicates of every donor (donor 10-12) and for respective THP1 derived M0 batches used for co-cultures, measured in duplicates in the ELISA. Significances are given when DIC or APAP resulted in upregulation of cytokines in the cultures. hepM = hepatic macrophages. Relevant statistical differences are given as * p < 0.05, ** p < 0.01, **** p < 0.0001
